# MSAO-EDA: A Modified Snow Ablation Optimizer by Hybridizing with Estimation of Distribution Algorithm

**DOI:** 10.3390/biomimetics9100603

**Published:** 2024-10-07

**Authors:** Wuke Li, Xiaoxiao Chen, Hector Chimeremeze Okere

**Affiliations:** International College, Hunan University of Arts and Science, Changde 415000, China; liwuke@huas.edu.cn (W.L.); okerejunior@gmail.com (H.C.O.)

**Keywords:** snow ablation optimizer, hybridization, collaborative search framework, global optimization, CEC 2017 test suite, CEC 2022 test suite

## Abstract

Metaheuristic algorithms provide reliable and effective methods for solving challenging optimization problems. The snow ablation algorithm (SAO) performs favorably as a physics-based metaheuristic algorithm. Nevertheless, SAO has some shortcomings. SAO is overpowered in its exploitation, has difficulty in balancing the proportion of global and local search, and is prone to encountering local optimum traps when confronted with complex problems. To improve the capability of SAO, this paper proposes a modified snow ablation algorithm hybrid distribution estimation algorithm named MSAO-EDA. In this work, a collaborative search framework is proposed where SAO and EDA can be organically integrated together to fully utilize the exploitation capability of SAO and the exploration capability of EDA. Secondly, an offset EDA approach that combines the optimal solution and the agent itself is used to replace SAO’s exploration strategy for the purpose of enhancing SAO’s exploration capability. Finally, the convergence of SAO is accelerated by selecting the next generation of agents through a greedy strategy. MSAO-EDA is tested on the CEC 2017 and CEC 2022 test suites and compared with EO, RIME, MRFO, CFOA, and four advanced algorithms, AFDBARO, CSOAOA, EOSMA, and JADE. The experimental results show that MSAO-EDA has excellent efficiency in numerical optimization problems and is a highly competitive SAO variant.

## 1. Introduction

Optimization exists in every aspect of our daily life and fundamentally revolves around identifying the best choice from a number of alternatives. Generally, there are three basic elements which comprise any optimization problem: the decision variables, the objective function, and the constraints. The main objective of optimization is to determine the best values for a set of parameters so as to optimize the objective function (extremum) within the specified constraints. Optimization algorithms can be classified into two categories based on the process of the optimization problem. The first category is deterministic algorithms that rely on the analytic properties of the function. These algorithms are also known as exact optimization algorithms. Examples of this category include Newton’s method [1], the hill-climbing method [2], the branch and bound method [3], and linear programming [4]. These algorithms usually utilize mathematical analysis by calculating the derivative of the objective function and performing iterative search using gradient information. Deterministic algorithms perform well with linear, continuous, differentiable, and convex problems, but they encounter challenges when faced with real-world problems characterized by large scale, multiple decision variables, and the presence of discrete and integer constraints [5]. Optimization models for such problems may not be expressed analytically. In addition, certain real-world problems prioritize the need for real-time optimization, where obtaining an approximate solution is sufficient, rather than pursuing an optimal solution. To address this challenge, stochastic methods, including metaheuristics, have been developed and have become the method of choice for solving these complex problems. Metaheuristic algorithms differ from traditional methods in that they are able to perform global searches in complex problem spaces without the need for gradient information and strike a balance between exploration and exploitation [6]. These algorithms originate from the simulation of natural laws, biological population phenomena, or human behavior, and the optimal value of the problem can be solved by means of an objective function value and a small number of parameters setting. Metaheuristic algorithms can be categorized into evolution-based algorithms, swarm-based algorithms, physics-based algorithms, and human-based algorithms [7].

Evolution-based algorithms simulate biological evolution to solve problems based on the natural process of evolution. It guides the search for the optimal solution through selection, crossover, and mutation operations based on agent fitness, and gradually optimizes the agents in the continuous iteration to find the optimal solution of the problem. Common evolutionary algorithms include the Genetic Algorithm (GA) [8], Differential Evolution (DE) [9], Biogeography-Based Optimization (BBO) [10], Evolution Strategies (ES) [11], and the Estimation of Distribution Algorithm (EDA) [12], among others.

Swarm-based algorithms are a type of metaheuristic algorithm proposed by simulating the laws of biological populations, including insects, microorganisms, fish, and herds. Particle Swarm Optimization (PSO) [13] is one of the most classical swarm intelligence algorithms, which mainly simulates the process of bird foraging. Birds can adjust their search direction by sensing the optimal food position explored by agents and the optimal food position explored by the population so far. The Cuckoo Search (CS) [14], Artificial Bee Colony (ABC) [15], and Firefly Algorithm (FA) [16] are other SAs that mimic brood parasitism, bee colonies, and the flashing patterns of fireflies, respectively. Similar algorithms of this category also include Crested Porcupine Optimizer (CPO) [17], Grey Wolf Optimizer (GWO) [18], Spider Wasp Optimizer (SWO) [19], Tuna Swarm Optimization (TSO) [20], Snake Optimizer (SO) [21], Genghis Khan Shark Optimizer (GKSO) [22], African Vulture Optimization Algorithm (AVOA) [23], and others.

The human-based algorithms are inspired by the social behavior of humans. Kumar et al. introduced the Social Evolution and Learning Optimization (SELO) [24] based on human sociological learning behavior. The Football Team Training Algorithm (FTTA) [25] is inspired by the behavior of players in soccer training sessions. Teaching Learning-Based Optimization (TLBO) [26], Brain Storm Optimization (BSO) [27], the Rider Optimization Algorithm (ROA) [28], and the Preschool Education Optimization Algorithm (PEOA) [29] are also examples of human-based algorithms.

Physics-based algorithms are inspired by physical phenomena such as electromagnetic forces, gravity, and temperature variations. Since they may be based on theorems and formulas from specific fields, it is easier to prove their convergence than other algorithms. One of the most classic examples is the Simulated Annealing (SA) [30], proposed by N. Metropolis et al., based on the solid-state annealing principles in metallurgy. Similar algorithms of this category also include the Gravitational Search Algorithm (GSA) [31], Sine Cosine Algorithm (SCA) [32], Arithmetic Optimization Algorithm (AOA) [33], Weighted Mean of Vectors (INFO) [34], Kepler Optimization Algorithm (KOA) [35], and Energy Valley Optimizer (EVO) [36].

Numerous metaheuristic algorithms have designed by applying different constituent parts, but according to the No Free Lunch (NFL) theory [37], none of them is superior to the others in solving all optimization problems. In other words, a particular metaheuristic may show very promising results on one set of problems, but the same algorithm may show poor performance on another set of problems. Clearly, the NFL theory keeps this area of research very active, which results in new metaheuristics and improvements to current methods being proposed every year.

The snow ablation optimizer (SAO) is a metaheuristic optimization method based on natural heuristics, proposed by Deng and Liu [38]. The melting and sublimation of snow are simulated to find the optimal solution to complex problems. The validity of SAO is tested in their work. Compared with other metaheuristic algorithms, SAO has a more flexible structure and fewer parameters. However, SAO also has the disadvantages of low convergence accuracy, little population diversity, and premature convergence. Jia et al. proposed a heat transfer strategy and a condensation strategy to improve the deficiencies of the original two-population mechanism and enhance the optimization performance of SAO [39]. Xiao et al. proposed a multi-strategy improved SAO variant incorporating an initialization strategy, a greedy strategy, a differential strategy, and an inverse learning strategy [40]. Zhang et al. used chaotic mapping, an adaptive t-distribution learning strategy, and a reverse learning strategy to improve the ability of SAO to solve optimization problems [41]. The existing literature on improvement methods for SAO focuses on replacing or improving the original strategy. Another approach for improvement methods for metaheuristic algorithms is to hybridize multiple algorithms. In this paper, a modified snow ablation optimizer by hybridizing with estimation of distribution algorithm (MSAO-EDA) is proposed. In MSAO-EDA, the exploitation capability of SAO and exploration capability of EDA are fully utilized. We propose a collaborative search framework based on SAO and EDA and utilize a greedy strategy to fully retain high-quality candidate agents. An offset EDA method is introduced in the exploitation phase of SAO to fully guide the population evolution towards more promising trends and assist the algorithm to jump out of the local optimum trap more efficiently. The main contributions of this study are as follows.

(1) A cooperative search framework is proposed for fully utilizing the exploitation capability of SAO and the exploration capability of EDA.
(2) An offset EDA method is proposed and applied in the exploitation phase of SAO to steer the population towards more promising regions and push the algorithm away from local optima.
(3) Forty-one functions from the CEC 2018 test suite and the CEC 2022 test suite are employed to comprehensively examine the accuracy and robustness of the proposed MSAO-EDA. Eight basic or improved algorithms are chosen for comparison with MSAO-EDA. The Wilcoxon test, Friedman test, and Nemenyi test were used to comprehensively analyze the differences between MSAO-EDA and competitors.

The paper is organized as follows. Section 2 presents a brief introduction to the snow ablation algorithm, outlining its basic principles and mathematical modeling. Subsequently, Section 3 explains the details of our proposed MSAO-EDA. This includes the cooperative search framework, the greedy strategy, and the offset EDA method. This part provides a comprehensive description of MSAO-EDA with flowcharts and pseudo-code, and the time complexity analysis is also shown in this section. In Section 4, simulation experiments are conducted to provide an in-depth analysis of the performance of MSAO-EDA on various optimization problems. Finally, we summarize the work of this paper and discuss several directions for future research.

## 2. Principle of the Snow Ablation Optimizer

SAO is a physics-based metaheuristic algorithm inspired by the two processes that turn snow into liquid water and steam: melting and sublimation, and the evaporation process that turns liquid water directly into steam. SAO is constructed with four components to find the optimal solution: the initialization phase, the exploration phase, the exploitation phase, and the dual-population mechanism. In this section, the details of the SAO formula are shown separately.

### 2.1. Initialization Phase

The iterative process of SAO starts with randomly generated populations. The whole population is represented by a matrix with a structure of N rows and D columns, as shown in Equation (1). Here, N denotes the total number of agents and D denotes the dimension of the solution space. The upper and lower boundaries of the search domain are ub and lb.

(1) $$X=lb+rand\times \left(ub-lb\right)={\left(\begin{array}{cccccc} x_{1,1} & \ldots & x_{1,j} & x_{1,j+1} & \ldots & x_{1,D}\\ \vdots & \ddots & \vdots & \vdots & \ddots & \vdots \\ x_{i,1} & \cdots & x_{i,j} & x_{i,j+1} & \cdots & x_{i,D}\\ x_{i+1,1} & \ldots & x_{i+1,j} & \ddots & \ldots & x_{i+1,D}\\ \vdots & \ddots & \vdots & \vdots & \ddots & \vdots \\ x_{N,1} & \cdots & x_{N,j} & x_{N,j+1} & \cdots & x_{N,D} \end{array}\right)}_{N\times D}$$

where rand are numbers randomly generated between 0 and 1. The ith search agent position can be described as $X_{i}=\left(\begin{array}{ccccc} x_{i,1} & \ldots & x_{i,j} & \ldots & x_{i,D} \end{array}\right)$. Different candidate solutions correspond to different problem function values. These values are expressed through Equation (2).

(2) $$F={\left[F\left(X_{1}\right),F\left(X_{2}\right),\ldots ,F\left(X_{N}\right)\right]}_{1\times N}$$

In metaheuristic algorithms, the quality of a candidate solution is evaluated by the function value of the problem. Therefore, the agent corresponding to the best objective function value is considered as the best agent. In this paper, it is assumed that the minimization problem is solved, and hence, the agent with the minimum value of F is the best agent.

### 2.2. Exploration Phase

In the exploration phase, SAO simulates the transition of snow and liquid water to steam to achieve an exploration of the problem space. The motion of SAO agents obeys a normal distribution with mean 0 and variance 1. Each SAO agent updates its current position according to Equation (3).

(3) $$X_{i}^{t+1}=X_{elite}^{t}+BM_{i}\times \left(r_{1}\times \left(X_{best}^{t}-X_{i}^{t}\right)+\left(1-r_{1}\right)\times \left(X_{mean}^{t}-X_{i}^{t}\right)\right)$$

where $X_{i}^{t+1}$ represents the position of the ith agent in the iteration of t+1 and $BM_{i}$ is a set of random numbers with Brownian motion symbols. $r_{1}$ is the randomly generated value between 0 and 1, and $X_{best}^{t}$ is the best solution with minimum fitness obtained so far. t and $t_{\max}$ are the current and maximum iterations, respectively. In addition, $X_{mean}^{t}$ represents the current average location of the overall population, and $X_{elite}^{t}$ represents random agents selected from the elite pool, calculated as Equations (4) and (5).

(4) $$X_{mean}^{t}=\frac{\sum_{i=1}^{N}X_{i}^{t}}{N}$$

(5) $$X_{elite}^{t}\in \left[X_{best}^{t},X_{second}^{t},X_{third}^{t},X_{centroid}^{t}\right]$$

(6) $$X_{centroid}^{t}=\frac{\sum_{i=1}^{N_{a}}X_{i}^{t}}{N_{a}}$$

where $X_{second}^{t}$ and $X_{third}^{t}$ denote the second- and third-best agents in the population at the tth iteration. $X_{centroid}^{t}$ denotes the centroid position of the top half of agents in terms of fitness. $N_{a}$ denotes the number of half-populations, satisfying $N_{a}=N/2$.

### 2.3. Exploitation Phase

The exploitation phase of SAO motivates searching agents to focus on exploitation around the current optimal agent. This phase simulates the transition of snow to liquid water, which is a process modeled using the degree-day method [42], represented below.

(7) $$M^{t}=\left(0.35+0.25\times \frac{e^{\frac{t}{t_{\max}}}-1}{e-1}\right)\times e^{\frac{-t}{t_{\max}}}$$

The position update equation for this stage is Equation (7).

(8) $$X_{i}^{t+1}=M^{t}\times X_{best}^{t}+BM_{i}\times \left(r_{2}\times \left(X_{best}^{t}-X_{i}^{t}\right)+\left(1-r_{2}\right)\times \left(X_{mean}^{t}-X_{i}^{t}\right)\right)$$

where $r_{2}$ is a random number between −1 and 1.

### 2.4. Dual-Population Mechanism

The performance of metaheuristic algorithms depends heavily on the coordinated switching and balancing of exploitation and exploration behaviors. On the other hand, whether a metaheuristic algorithm performs well or poorly also depends on specific problem characteristics, as pointed out by the NFL theory. In SAO, a dual-population mechanism is proposed to maintain the exploitation and exploration behaviors. In this mechanism, the population is initially divided into two equal-numbered sub-populations, $Pop_{a}$ and $Pop_{b}$. The number of agents in these two sub-populations are $N_{a}$ and $N_{b}$. As the search progresses, the number of $N_{a}$ increases and the number of $N_{b}$ correspondingly decreases.

(9) $$N_{a}=N_{a}+1,N_{b}=N_{b}-1$$

As presented in Algorithm 1, *N_a_* is increased by 1 and $N_{b}$ is decreased by 1 after each iteration when the stagnation criterion is not met.
**Algorithm 1** Pseudo-code of dual-population mechanism1:  Data: Population size N, iteration t, maximum iteration $t_{\max}$, dimension size D, $N_{a}=N_{b}=0.5N$ 2:  **While** $t<t_{\max}$ 3:    **If** $N_{a}<N$ 4:    $N_{a}=N_{a}+1,N_{b}=N_{b}-1$ 5:    **End if** 6:    t=t+1 7:  **End while**

## 3. The Proposed MSAO-EDA

SAO is rather effective in accurately exploiting the problem space, but it suffers from several drawbacks, i.e., imbalance in exploration and exploitation capabilities, lack of population diversity, and tendency to descend into local optima. Jia et al. point out that the SAO algorithm has a reduced exploitation capability in the later iterations due to the conversion of all snow to gas [39]. Xiao et al. pointed out that SAO exhibits weak convergence performance in the face of high-dimensional problems [40]. The starting point of this work is to address the shortcomings of SAO, for which we propose MSAO-EDA. In our work, SAO and EDA are hybridized by proposing a collaborative search framework that is able to utilize the respective advantages of both algorithms. Secondly, we use an offset EDA method to replace the exploitation strategy of SAO in this paper to further enhance the population diversity. In this section, an overview of EDA will be given first, and then we will give the details of the collaborative search framework in Section 3.2. Based on EDA, we propose the offset EDA method in Section 3.3. Finally, the flowchart and pseudo-code of the proposed MSAO-EDA are presented, and time complexity analysis is performed.

### 3.1. Estimation of Distribution Algorithm

EDA is an evolution-based metaheuristic algorithm [12]. EDA performs iterative evolution of the entire candidate population by modeling the probability distribution of the candidate solutions. In this section, the basics of EDA will be briefly introduced, and its optimization process is roughly shown below.

(1) Initializing the candidate population.
(2) Calculating the fitness of the population and updating the global best candidate agent.
(3) Selecting some dominant agents to create a Gaussian distribution probability model.
(4) Generating new agents by sampling based on the probability distribution model.
(5) If the algorithm terminates, output the optimal agent; otherwise, execute (2).

The Gaussian distribution probability model in Step 3 is the most widely used probability model for EDA. The joint Gaussian probability density function of a *D*-dimensional random vector can be expressed as Equation (10).

(10) $$G{\left(X\right)}_{\mu ,C}=\sqrt{\frac{1}{{\left(2\times \pi \right)}^{D}\times \det\left(C\right)}}\times e^{\left(-{\left(X-\mu \right)}^{T}\times C^{-1}\times \left(X-\mu \right)/2\right)}$$

(11) $$\mu =\frac{1}{N_{d}}\times \sum_{i=1}^{N_{d}}X_{i},X_{i}\in P_{d}$$

(12) $$C=\frac{1}{N_{d}}\times \sum_{i=1}^{N_{d}}\left(X_{i}-\mu \right)\times {\left(X_{i}-\mu \right)}^{T},X_{i}\in P_{d}$$

where $N_{d}$ denotes the number of dominant agents included in $P_{d}$. Dominant agents denote agents ranking in the top half of fitness. Once the Gaussian probability distribution model of the dominant population is obtained, the population is sampled according to a mean of 1 and a covariance matrix of C to generate offspring.

(13) $$X_{i}=\mu +g_{i},g_{i}\sim N\left(0,C\right)$$

### 3.2. Collaborative Search Framework

**Figure 1:**
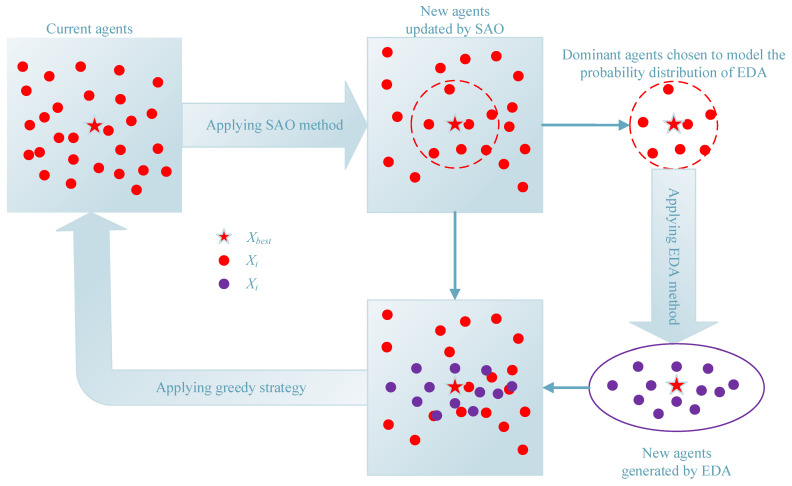
Sketch for procedure of collaborative search framework.

As shown in the introduction, the research on hybrid algorithms is an important direction to improve the performance of an algorithm. Existing research on SAO mainly focuses on the modification of the original search method and initialization improvement, as well as the incorporation of additional search methods, such as reverse learning strategies, mutation strategies, etc. In this work, MSAO-EDA uses a novel collaborative search framework to fully utilize the strong local search capability of SAO and the global exploration capability of EDA. Specifically, SAO absorbs the information of the optimal agent in both its exploitation and exploration phases, which can weaken the population diversity of SAO, and thus is prone to fall into the local optimum trap. EDA fully adopts the valid information of multiple agents by sampling the dominant population and exhibits a strong exploration capability. Considering the characteristics of SAO and EDA, the collaborative search framework implements the exchange of population information between the two methods. The details are shown in Figure 1. The two methods are executed in series. Firstly, SAO is used to update the population, and then EDA is used to select some dominant agents from the SAO-updated population to model the probability distribution. Then new agents are generated by sampling based on the established probability distribution model. Finally, a greedy strategy is used to select better N agents from the SAO-updated agents and EDA-generated agents for the next iteration of update.

In MSAO-EDA, the number $N_{d}$ is half the number of populations. This provides a more accurate Gaussian probability distribution model. Regarding the number of agents $N_{E}$ generated by the EDA method: considering that too much $N_{E}$ will waste the number of fitness evaluations and too little $N_{E}$ cannot perform the role of EDA, this paper proposes an adaptive $N_{E}$ adjustment as shown in Equation (14).

(14) $$N_{E}=\rho \times \left(0.35+0.15\times \frac{t}{t_{\max}}\right)$$

where ρ is used to control the number of $N_{E}$; the exact value will be discussed in the experimental section.

### 3.3. Offset EDA Strategy

**Figure 2:**
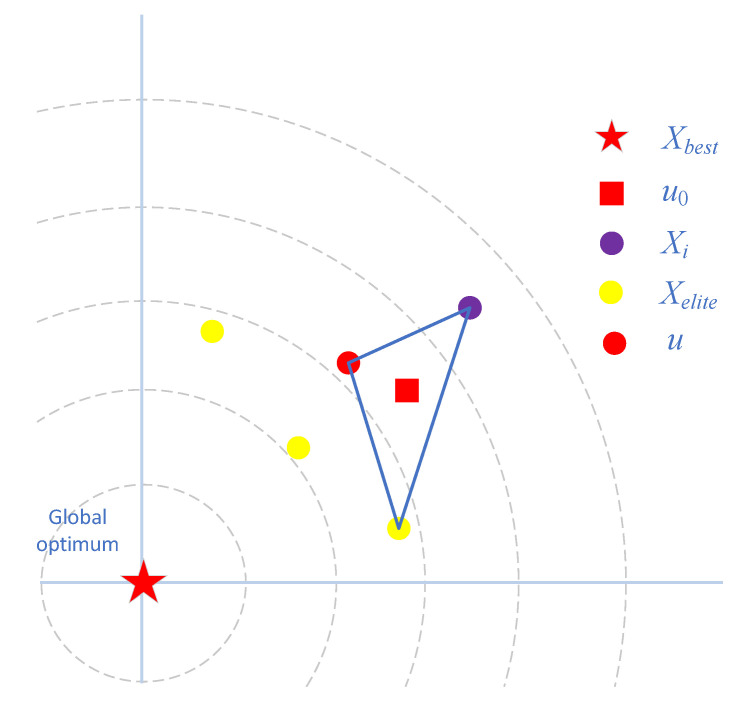
Sketch for procedure of Offset EDA strategy.

SAO over-absorbs the location information of the optimal solution and the three sub-optimal solutions in the exploration phase, which can weaken the population diversity of SAO. Therefore, this paper considers incorporating the EDA method into the exploration strategy of SAO. Furthermore, due to the existence of the dual population mechanism, SAO will carry out more exploration behaviors in the later phase of the optimization, which requires that the exploration strategy also needs to have some exploitation capability, thus ensuring the convergence of SAO. The agents generated by the EDA method have an important connection with the mean μ. The literature [43] proposes a multileader-based search diversification strategy that corrects the mean value μ using the dominant solution and the agent itself, respectively. In this paper, inspired by the literature [43], we propose an offset EDA method (OEDA). In OEDA, the agents adjust the mean value using both their own position information and some of the elite solution position information. This enables a more comprehensive exploration of the problem space, maintains population diversity to avoid the algorithm from stagnating, and also accelerates the convergence of the algorithm. A schematic of the OEDA method is shown in Figure 2. The OEDA is shown in Equation (15).

(15) $$X_{i}=\mu_{O}+g_{i},g_{i}\sim N\left(0,C\right)$$

(16) $$\mu_{O}=X_{i}+\mu +X_{elite}^{t}$$

For each agent executing exploration behavior, if $rand<t/t_{\max}$, the SAO original search method is executed; otherwise, the OEDA method is employed. This selection ensures that OEDA is utilized more in the early stages to correctly orient the population movement to more promising areas and enhance population diversity. The SAO original strategy is used more often in the later stages to carry out local exploitation and accelerate SAO convergence.

In MSAO-EDA, the OEDA strategy and the SAO original strategy are applied in the exploration population during the SAO phase. The EDA phase follows on from the completion of the SAO phase. New agents produced by both phases are selected by the greedy strategy. In summary, the pseudo-code of MSAO-EDA is shown in Algorithm 2.
**Algorithm 2** Pseudo-code of MSAO-EDA1:  Data: Population size N, current fitness evaluation number FEs, maximum fitness evaluation number $FEs_{\max}$, dimension size D, $N_{a}=N_{b}=0.5N$ 2:  Result: Global optimal solution $X_{best}^{t}$3:  Initialize the random population X using Equation (1).
4:  Evaluate X to determine their fitness value by using $F\left(X\right)$.  5:  While $FEs<FEs_{\max}$
6:    **Applying SAO method**
7:    Calculate the snowmelt ratio using Equation (7). 8:    Randomly split the entire population into two sub-populations: $P_{a}$ and $P_{b}$. 9:    **For** each agent $X_{i}$ in $P_{a}\left(i=1,2,\ldots ,N_{a}\right)$ do 10:      **If** $rand<(FEs/FEs_{\max})$ 11:         Update the position of the ith agent using Equation (3). 12:       **Else**
13:        Update the position of the ith agent using Equation (15). ||**Offset EDA strategy**
14:     **End if** 15:      **If** $N_{a}<N$ 16:        $N_{a}=N_{a}+1,N_{b}=N_{b}-1$
17:     **End if**
18:    **For** each agent $X_{i}$ in $P_{b}\left(i=1,2,\ldots ,N_{b}\right)$ do 19:      Update the position of the ith agent using Equation (8). 20:    **End if**
21:    Evaluate X to determine their fitness value by using $F\left(X\right)$. 22:    FEs=FEs+N
23:    **Applying EDA method** 24:    Selecting the dominant agents for EDA 25:    Estimate $G{\left(X\right)}_{\mu ,C}$ by using Equations (11) and (12). 26:    Generating new agents $X_{i}\left(i=1,2,\ldots ,N_{d}\right)$ using Equation (13) to form $P_{d}$. 27:    Evaluate X to determine their fitness value by using $F\left(X\right)$. 28:    $FEs=FEs+N_{d}$ 29:    Selecting better agents from $P_{a}$, $P_{b}$ and $P_{d}$ using greedy strategy. 30:  **End while**

### 3.4. Time Complexity Analysis of MSAO-EDA

It is necessary to consider the time complexity for the optimization algorithm. There are three main influencing factors to consider: population size N, dimension of optimization problem D, and number of maximum iterations $t_{\max}$. For the basic SAO, the time complexity of initialization process is $O\left(N\times D\right)$. Then, in the iterative process, the time complexity is $O\left(N_{a}\times D\times T+N_{b}\times D\times T\right)$. Therefore, the total time complexity of SAO is as below.

$$Comp\left(SAO\right)=O\left(N_{a}\times D\times T+N_{b}\times D\times T+N\times D\right)≅O\left(N\times D\times T\right)$$

In the proposed MSAO-EDA, the Offset EDA strategy is used in the exploitation phase of SAO in cooperation with the original exploitation strategy for population updates and therefore does not affect the time complexity. The EDA method is combined with SAO through the collaborative search framework, and $N_{d}$ agents are generated in this phase, so the time complexity of this part is $O\left(N_{d}\times D\times T\right)$. Therefore, the total time complexity of MSAO-EDA is as below.

$$Comp\left(MSAO‐EDA\right)=O\left(N_{a}\times D\times T+N_{b}\times D\times T+N\times D+N_{d}\times D\times T\right)≅O\left(\left(N+N_{d}\right)\times D\times T\right)$$

## 4. Analysis of Experiments and Results

In this section, we designed three different experiments to evaluate the performance of the proposed MSAO-EDA algorithm. First, we discussed the sensitivity of the MSAO-EDA to parameter ρ and evaluated the effectiveness of the improved method. Then, comparisons were made with eight state-of-the-art algorithms on the CEC2017 and CEC2022 benchmark test suites. Subsequently, we subjected it to the Wilcoxon rank sum test and Friedman test to ascertain whether significant performance discrepancies exist between MSAO-EDA and other algorithms.

### 4.1. Experimental Settings

All optimization methods were programmed by MATLAB R2021a and implemented in Lenovo Legion Y9000P, which is equipped with Windows 10, 14th Intel^(R)^ Core^(TM)^ i9-14900HX, and 32 GB RAM.

Table 1 and Table 2 present the specific details of these two test suites. It can be observed that the CEC2017 test functions contain twenty-nine functions with four types, including unimodal functions (F1, F2), simple multimodal functions (F3–F9), hybrid functions (F10–F19), and composition functions (F20–F29), while the CEC2022 test functions have twelve functions with four types, including unimodal functions (F1), simple multimodal functions (F2–F5), hybrid functions (F6–F8), and composition functions (F9–F12). The search range for both test suites is [−100,100] and the problems’ dimension is 10/30/50/100 for CEC2017 and 10/20 for CEC2022.

The results of MSAO-EDA are compared to eight optimization methods, including four standard algorithms, EO [44], RIME [45], MRFO [46], CFOA [8], and four advanced algorithms, AFDBARO [47], CSOAOA [48], EOSMA [49], JADE [50]. Table 3 presents the parameter settings of MSAO-EDA and other competitive algorithms. The maximum function evaluation ($FEs_{\max}$) is set to *D* × 10,000 for all benchmark functions. Each test function has been tested 51 times independently to eliminate the effect of randomness, and the statistical result includes the best, mean, and standard deviation of the value of the objective function.

### 4.2. Sensitivity of MSAO-EDA to Parameter ρ

**Figure 3:**
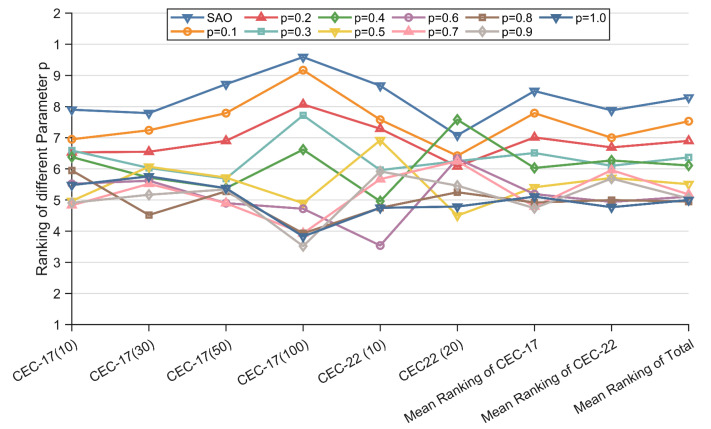
Rankings of the different parameter *ρ*.

For metaheuristic algorithms, the setting of parameters affects the performance of an algorithm in an important way. In order to fully utilize the performance of MSAO-EDA, the new parameter ρ introduced in MSAO-EDA needs to be discussed to obtain the optimal settings. In this paper, the performance of MSAO-EDA with different values of the parameter ρ is evaluated using the benchmark functions of the CEC 2017 test suite and the CEC2022 test suite. The values of the parameter ρ satisfy $\rho \in \left\{0.1,0.2,0.3,0.4,0.5,0.6,0.7,0.8,0.9,1.0\right\}$. The Friedman test scores of the results obtained for MSAO-EDA with different values of the parameter ρ are summarized in Table 4 and visualized in Figure 3. From Figure 3, it can be seen that the best parameter setting emerged at ρ=0.8, and the average score of MASO-EDA with this parameter setting was 4.95. Therefore, in this paper, the parameter ρ was set to 0.8 in order to fully demonstrate the performance of MSAO-EDA.

### 4.3. Strategies Effectiveness Analysis

In this section, we evaluate the impact of the collaborative search framework and offset EDA strategy on MSAO-EDA. Based on the principle of ablation experiments, two MSAO-EDA variants are proposed in this section, which apply the collaborative search framework and offset EDA strategy, i.e., MSAO-EDA-1 and MSAO-EDA-2. Details of MSAO-EDA and its variants are shown in Table 5. MSAO-EDA-2 is used to analyze the effect of the collaborative search framework. MSAO-EDA-1 reflects the effect of offset EDA strategy on the performance of SAO. Similarly, the Friedman test is employed to analyze the test results.

**Figure 4:**
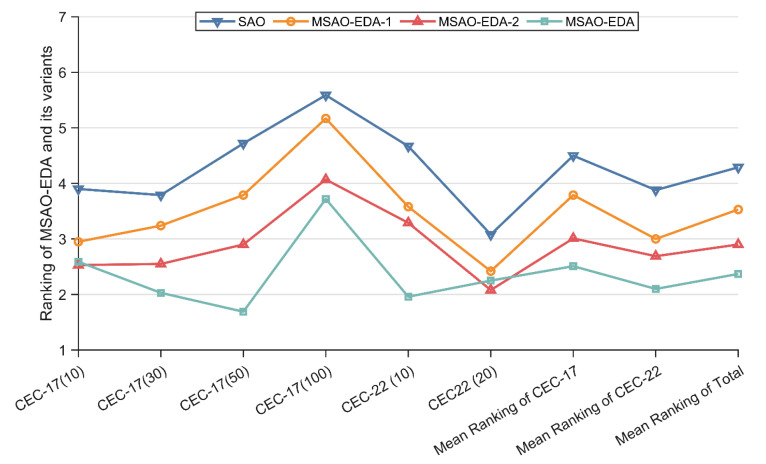
Rankings of MSAO-EDA and its variants.

Table 6 summarizes the Friedman test results on both test suites for MSAO-EDA, MSAO-EDA-1, MSAO-EDA-2, and SAO. As shown in Table 6, the Friedman *p*-values in the last column are all less than 0.05, which indicates that there is a significant difference in performance between MSAO-EDA, MSAO-EDA-1, MSAO-EDA-2, and SAO on all dimensions of the two test suites. Figure 4 visualizes the Friedman scores for each algorithm. The discussion based on Table 6, and Figure 4 is shown below.

The impact of offset EDA strategy on the performance of MSAO-EDA can be analyzed by comparing MSAO-EDA with MSAO-EDA-2. For the CEC2017 test suite, MSAO-EDA beats MSAO-EDA-2 on 30 D, 50 D, and 100 D, and loses ground to MSAO-EDA-2 on 10 D. For the CEC2022 test suite, MSAO-EDA beats MSAO-EDA-2 on 10 D and loses ground to MSAO-EDA-2 on 20 D. The performance of MSAO-EDA on the CECE2017 average scores, CEC2022 average scores, and total average scores is better than that of MSAO-EDA-2. Therefore, the collaborative search framework can effectively improve the performance of MSAO-EDA in 10/30/50 D in CEC2017 and 10 D in CEC2022.

The impact of the collaborative search framework on the performance of MSAO-EDA can be analyzed by comparing MSAO-EDA with MSAO-EDA-1. For the CEC2017 test suite, MSAO-EDA beats MSAO-EDA-1 on 10 D, 30 D, 50 D, and 100 D. For the CEC2022 test suite, MSAO-EDA beats MSAO-EDA-1 on 10 D and 20 D. The performance of MSAO-EDA on the CECE2017 average scores, CEC2022 average scores, and total average scores is better than that of MSAO-EDA-1. Therefore, the collaborative search framework can effectively improve the performance of MSAO-EDA in 10/30/50/100 D in the CEC2017 test suite and 10/20 D in the CEC2022 test suite.

Furthermore, MSAO-EDA is ranked first on CEC2017 mean rank, CEC2022 mean rank, and total mean rank. MSAO-EDA-2 and MSAO-EDA-1 are ranked second and third. SAO is ranked last on all cases. These analyses show that the improvement method proposed in this paper effectively boosts the performance of SAO.

### 4.4. Performance Analysis Based on CEC2017 Test Suite

**Figure 5:**
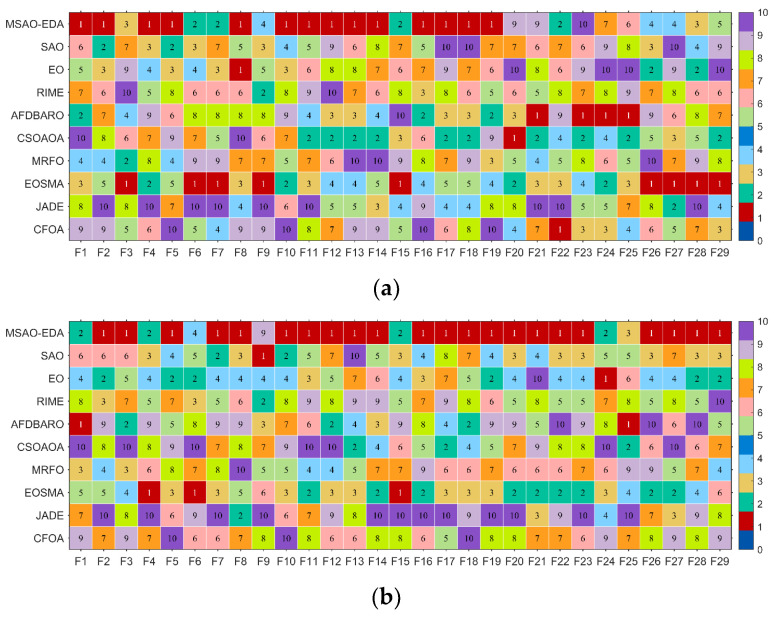

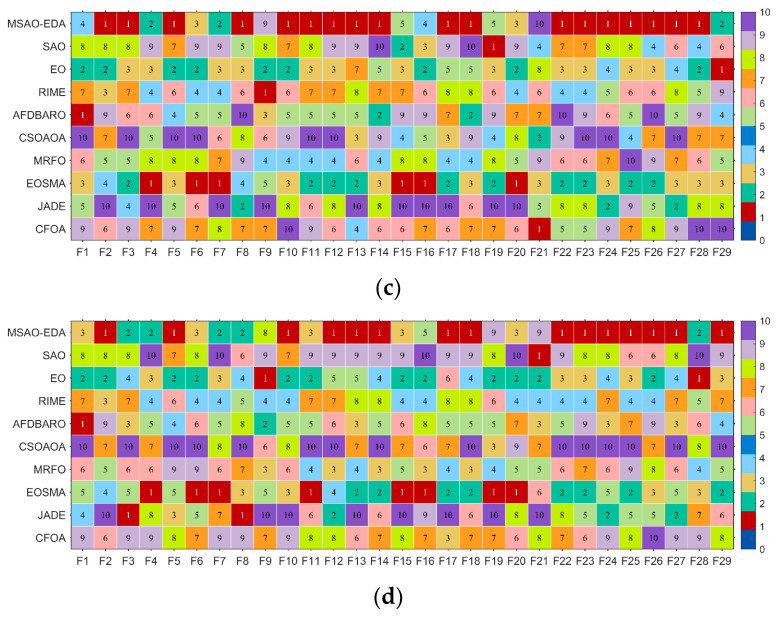
The rankings of MSAO-EDA and its competitors. (**a**) D = 10; (**b**) D = 30; (**c**) D = 50; (**d**) D = 100.

In this section, we evaluate the performance of the MSAO-EDA algorithm on the CEC2017 test suite. All parameter settings are the same as in Section 4.1. The Wilcoxon rank sum test, the Friedman test, the convergence analysis, and the stability analysis are used to evaluate the optimization ability of MSAO-EDA from various perspectives. Specific results containing optimal values, mean, standard deviation, and rankings of MSAO-EDA and the comparison algorithms on the CEC2017 test suite can be found in Table A1, Table A2, Table A3 and Table A4 in Appendix A. Firstly, we draw heat maps to present the performance of each algorithm on different benchmark functions based on the rankings in Table A1, Table A2, Table A3 and Table A4, as shown in Figure 5. We can roughly conclude that MSAO-EDA outperforms its competitors on 30 D, 50 D, and 100 D according to Figure 5.

#### 4.4.1. Analysis for the Wilcoxon Rank Sum Test Results

**Figure 6:**
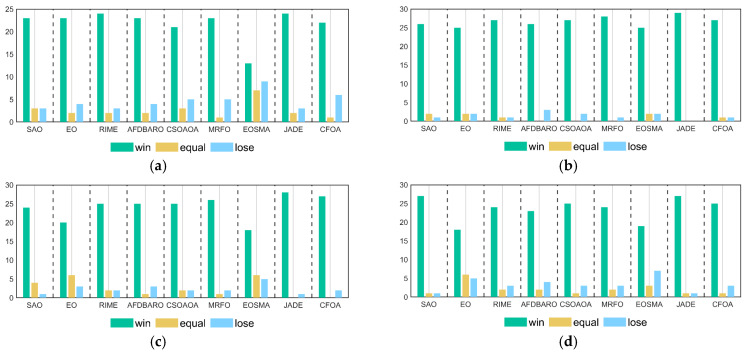
The Wilcoxon rank sum results of MSAO-EDA and its competitors on CEC2017. (**a**) D = 10; (**b**) D = 30; (**c**) D = 50; (**d**) D = 100.

In this section, Wilcoxon rank sum test is used to analyze the performance differences between MSAO-EDA and the comparison algorithms on different functions. Under the null hypothesis H0, it is assumed that there is no significant difference between the two algorithms. If the calculated *p*-value is less than 5%, the null hypothesis is rejected, indicating a substantial difference between the algorithms. Conversely, if the *p*-value exceeds 5%, the null hypothesis is supported, indicating a lack of significant difference and implying comparable performance between the algorithms. The detailed results of the Wilcoxon rank sum test with a significance level of a = 0.05 for MSAO-EDA and competitors on all dimensions using the CEC 2017 test suite are presented in Table A5, Table A6, Table A7 and Table A8 in Appendix A. Table 7 summarizes the numbers of MSAO-EDA significantly superior, similar, and inferior to those of competitors, indicated by “+/=/−”, respectively. Figure 6 visualizes the results of the Wilcoxon rank sum test for MSAO-EDA and the comparison algorithms under the CEC2017 test suite. The specific analysis is as follows.

For 10 D, MSAO-EDA bests (loses) SAO, EO, RIME, AFDBARO, CSOAOA, MRFO, EOSMA, JADE, and CFOA, on 23 (3), 23 (4), 24 (3), 23 (4), 21 (5), 23 (5), 13 (9), 24 (3), and 22 (6) benchmark test functions. Thus, MSAO-EDA significantly outperforms the comparison algorithms on 10 D.

For 30 D, MSAO-EDA bests (loses) SAO, EO, RIME, AFDBARO, CSOAOA, MRFO, EOSMA, JADE, and CFOA, on 26 (1), 25 (2), 27 (1), 26 (3), 27 (2), 28 (1), 25 (2), 29 (0), and 27 (1) benchmark test functions. Thus, MSAO-EDA significantly outperforms the comparison algorithms on 30 D.

For 50 D, MSAO-EDA bests (loses) SAO, EO, RIME, AFDBARO, CSOAOA, MRFO, EOSMA, JADE, and CFOA, on 24 (1), 20 (3), 25 (2), 25 (3), 25 (2), 26 (2), 18 (5), 28 (1), and 27 (2) benchmark test functions. Thus, MSAO-EDA significantly outperforms the comparison algorithms on 50 D.

For 100 D, MSAO-EDA bests (loses) SAO, EO, RIME, AFDBARO, CSOAOA, MRFO, EOSMA, JADE, and CFOA, on 27 (1), 18 (5), 24 (3), 23 (4), 25 (3), 24 (3), 19 (7), 27 (1), and 25 (3) benchmark test functions. Thus, MSAO-EDA significantly outperforms the comparison algorithms on 100 D.

Summarizing the above analysis, we can conclude that the performance of MSAO-EDA on the CEC2017 test suite is significantly better than those of SAO, EO, RIME, AFDBARO, CSOAOA, MRFO, EOSMA, JADE, and CFOA.

#### 4.4.2. Analysis for the Friedman Test Results

**Figure 7:**
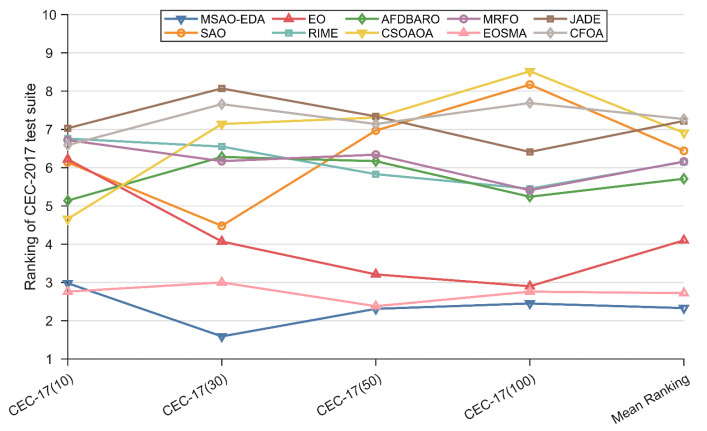
The Friedman test results of MSAO-EDA and its competitors on CEC2017.

In this section, the Friedman test is implemented to analyze the performance of MSAO-EDA and the competitors on the CEC2017 test suite with different dimensions. Table 8 summarizes the Friedman scores of these algorithms, which are visualized in Figure 7. According to Table 8, the Friedman *p*-values in the last column are all less than 0.05, which indicates that there is a significant difference between MSAO-EDA and competitors. The specific analysis is as follows.

For 10 D, MSAO-EDA is ranked second after EOSMA, and the performance of the remaining algorithms are ranked as CSOAOA, AFDBARO, SAO, EO, CFOA, MRFO, RIME, and JADE.

For 30 D, MSAO-EDA ranks first, followed by EOSMA, EO, SAO, MRFO, AFDBARO, RIME, CSOAOA, CFOA, and JADE.

For 50 D, MSAO-EDA ranks first, followed by EOSMA, EO, RIME, AFDBARO, MRFO, SAO, CFOA, CSOAOA, and JADE.

For 100 D, MSAO-EDA ranks first, followed by EOSMA, EO, AFDBARO, MRFO, RIME, JADE, CFOA, SAO, and CSOAOA.

In conclusion, the overall performance of MSAO-EDA on the CEC2017 test suite is the best, inferior to EOSMA only on 10 D, ranking first on the other three dimensions, and outperforming the basic SAO in all cases. Therefore, the results of the Friedman test support the fact that MSAO-EDA is a high-performance method.

#### 4.4.3. Analysis for the Convergence Ability and Stability

**Figure 8:**
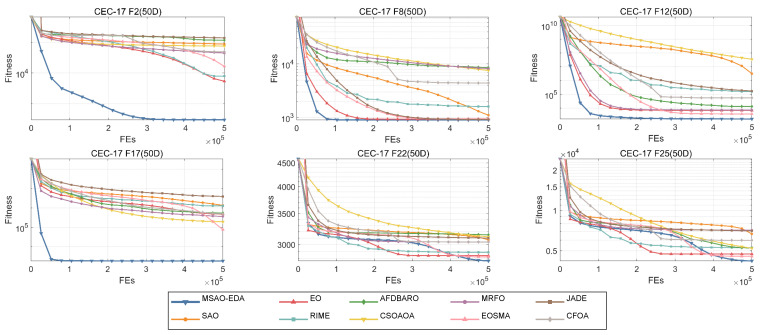
The convergence graphs of MSAO-EDA and its competitors on CEC2017 50 D.

**Figure 9:**
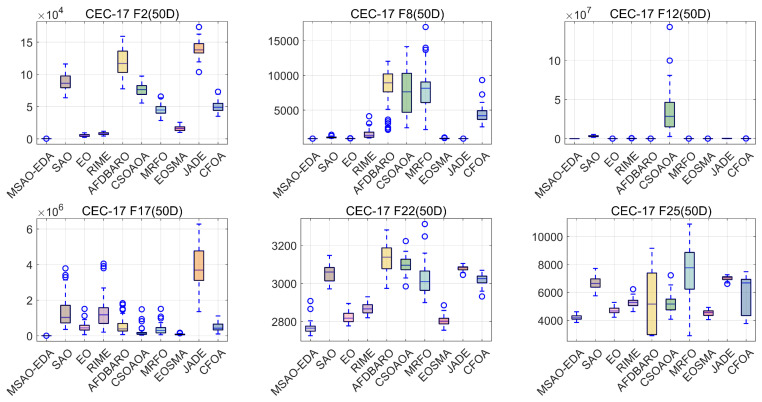
The box graph of MSAO-EDA and its competitors on CEC2017 50 D.

**Figure A1:**
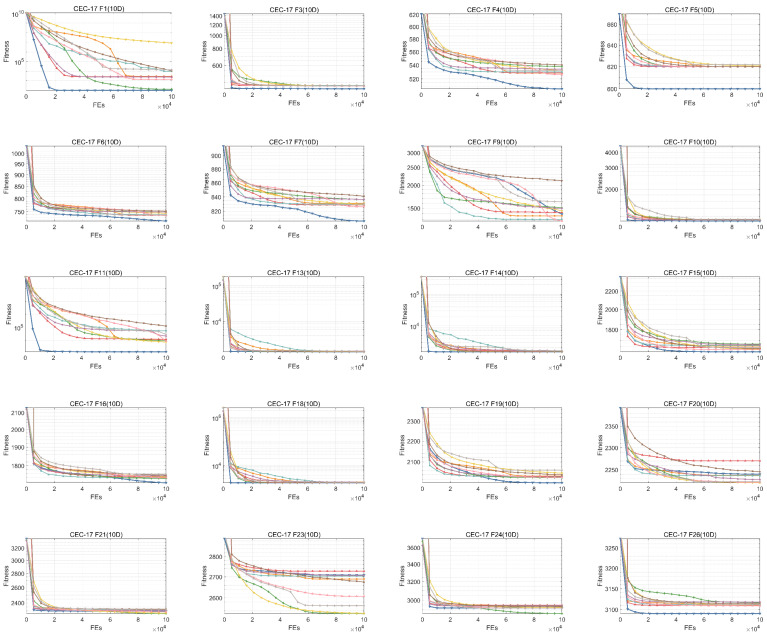

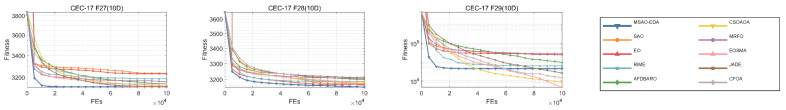
The convergence graphs of MSAO-EDA and its competitors on CEC2017 (10 D).

**Figure A2:**
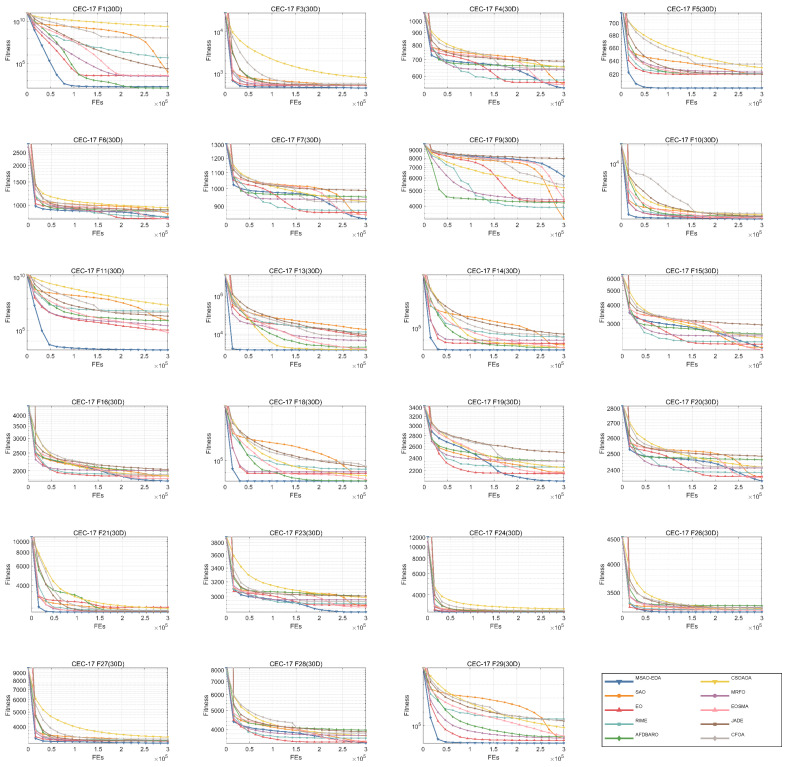
The convergence graphs of MSAO-EDA and its competitors on CEC2017 (30 D).

**Figure A3:**
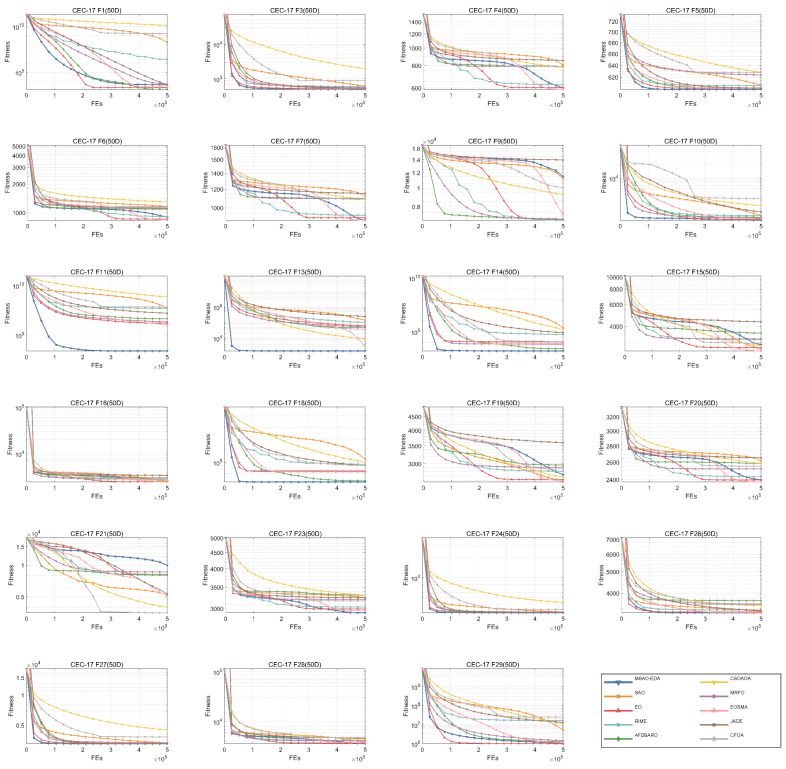
The convergence graphs of MSAO-EDA and its competitors on CEC2017 (50 D).

**Figure A4:**
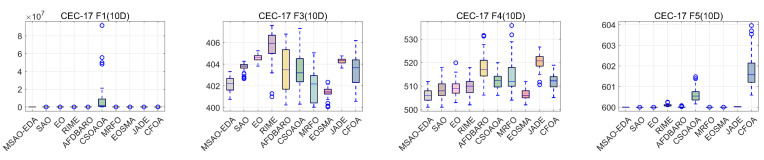

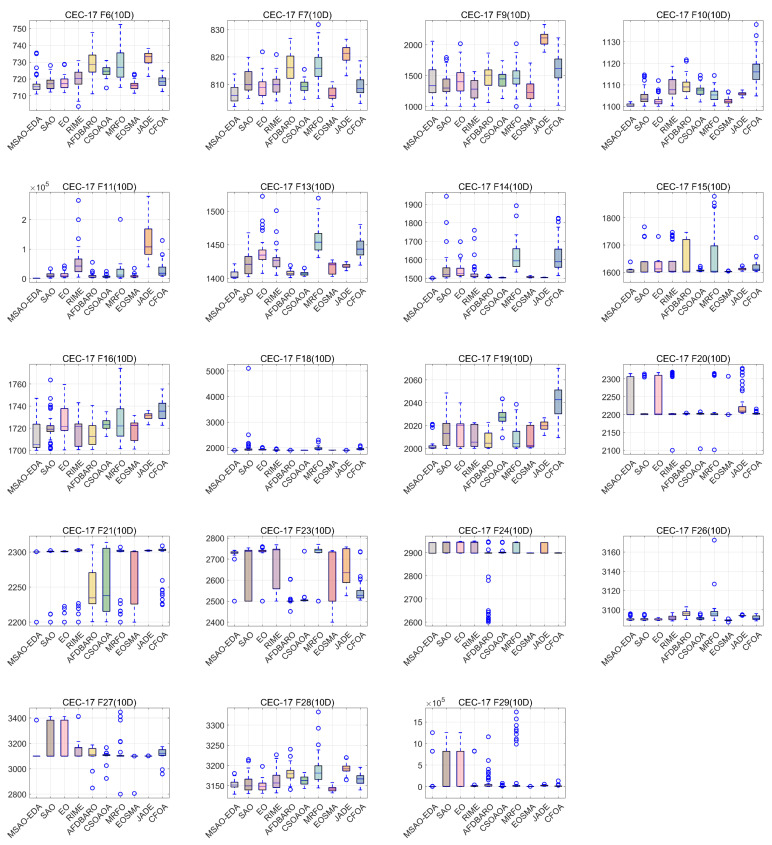
The box graphs of MSAO-EDA and its competitors on CEC2017 (10 D).

**Figure A5:**
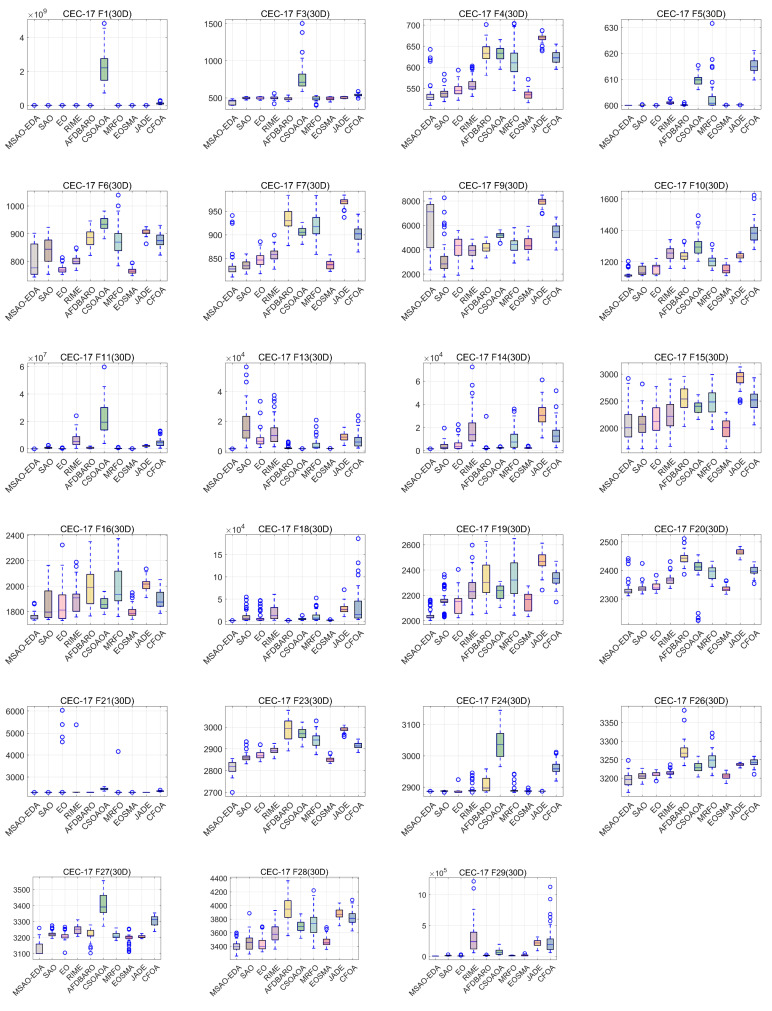
The box graphs of MSAO-EDA and its competitors on CEC2017 (30 D).

**Figure A6:**
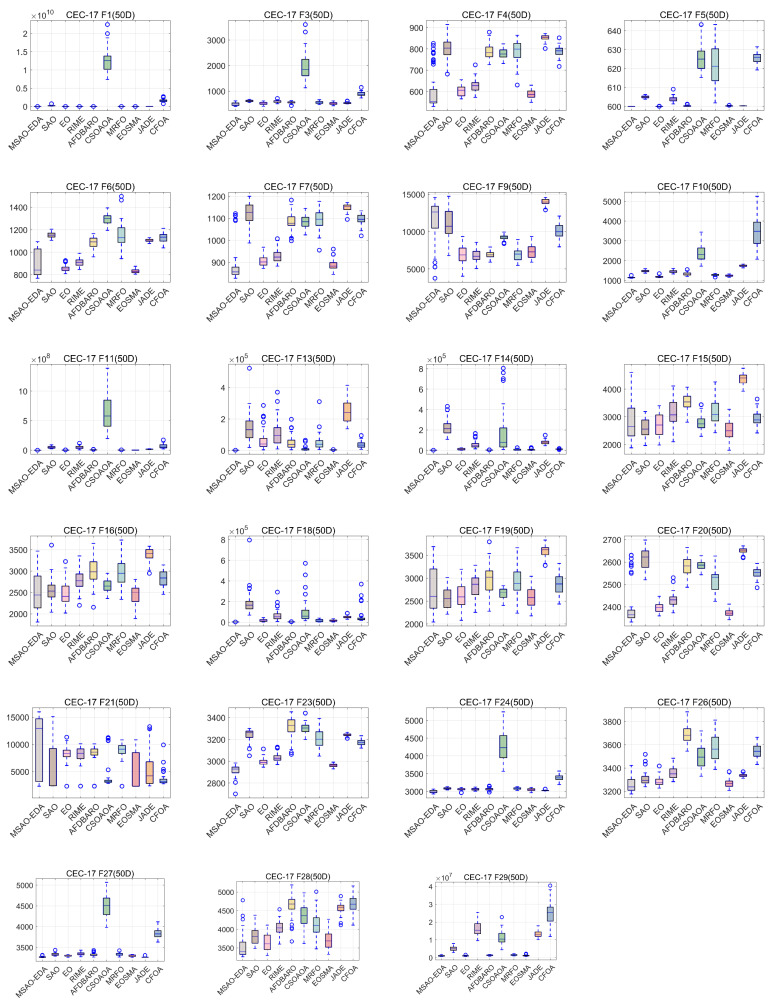
The box graphs of MSAO-EDA and its competitors on CEC2017 (50 D).

In this section, we will present the convergence performance and robustness of MSAO-EDA on the CEC2017 test suite. For the convenience of reading, the convergence plots and box plots of six 50 D functions (one unimodal function (F2), one multimodal function (F9), two hybrid functions (F12 and F17), and two composite functions (F22 and F25)) are displayed in Figure 8 and Figure 9. The complete convergence curves and box plots are available in Figure A1, Figure A2, Figure A3, Figure A4, Figure A5 and Figure A6 in Appendix B.

Notably, the blue line in Figure 8 denotes the MSAO-EDA proposed in this paper. According to Figure 8, it can be found that MSAO-EDA shows the fastest convergence speed and higher convergence accuracy on F2, F8, F12, and F17. For F22 and F25, MSAO-EDA converges more slowly in the early stage, but is able to get rid of the local optimum in the later stage and obtain a higher accuracy solution. This shows that MSAO-EDA outperforms the other compared algorithms in terms of convergence performance.

Figure 9 shows a box plot of the results obtained based on 51 runs. The symbol “o” in the figure indicates outliers. The upper and lower boundaries of the box indicate the extent of the distribution of solutions. The closer the upper and lower boundaries are, the more concentrated the solution distribution is. The overall height of the box reflects the overall quality of the solution. According to Figure 9, it can be seen that MSAO-EDA performs best in terms of the number of outliers, the size of the box, and the overall height, showing the best stability.

### 4.5. Performance Analysis Based on CEC2022 Test Suite

**Figure 10:**
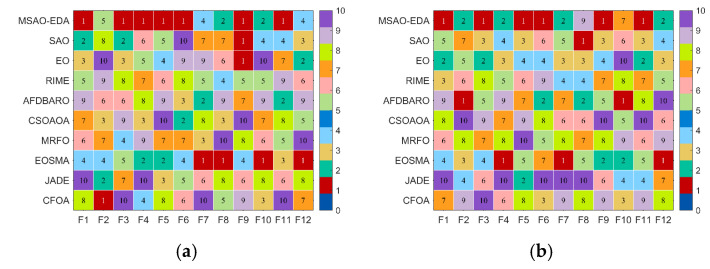
The rankings of MSAO-EDA and its competitors on CEC2022. (**a**) D = 10; (**b**) D = 20.

In this section, we evaluate the performance of the MSAO-EDA algorithm on the CEC2022 test suite. All parameter settings are the same as in Section 4.1. The Wilcoxon rank sum test, the Friedman test, the convergence analysis, and the stability analysis are used to evaluate the optimization ability of MSAO-EDA from various perspectives. Specific results containing optimal values, mean, standard deviation, and rankings of MSAO-EDA and the comparison algorithms on the CEC2022 test suite can be found in Table A9, Table A10, Table A11 and Table A12 in Appendix A. Firstly, we draw heat maps to present the performance of each algorithm on different benchmark functions based on the rankings in Table A9 and Table A10, as shown in Figure 10. We can roughly conclude that MSAO-EDA outperforms its competitors on 10 D and 20 D, according to Figure 10.

#### 4.5.1. Analysis for the Wilcoxon Rank Sum Test Results

**Figure 11:**
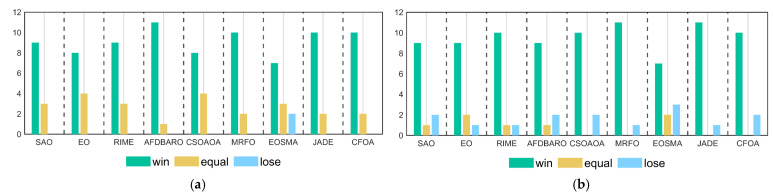
The Wilcoxon rank sum results of MSAO-EDA and its competitors on CEC2022. (**a**) D = 10; (**b**) D = 20.

In this section, the Wilcoxon rank sum test is used to analyze the performance differences between MSAO-EDA and its competitors on CEC2022 test functions. The detailed results of the Wilcoxon rank sum test with a significance level of a = 0.05 for MSAO-EDA and competitors on 10 D and 20 D using the CEC 2022 test suite are presented in Table A11 and Table A12 in Appendix A. Table 9 summarizes the numbers of MSAO-EDA significantly superior, similar, and inferior to those of competitors, indicated by “+/=/−”, respectively. Figure 11 visualizes the results of the Wilcoxon rank sum test for MSAO-EDA and the comparison algorithms under the CEC2022 test suite. The specific analysis is as follows.

For 10 D, MSAO-EDA is superior (inferior) SAO, EO, RIME, AFDBARO, CSOAOA, MRFO, EOSMA, JADE, and CFOA, on 9 (0), 8 (0), 9 (0), 11 (0), 8 (0), 10 (0), 7 (2), 10 (0) and 10 (0) benchmark test functions. Thus, MSAO-EDA significantly outperforms the comparison algorithms on 10 D.

For 20 D, MSAO-EDA is superior (inferior) SAO, EO, RIME, AFDBARO, CSOAOA, MRFO, EOSMA, JADE, and CFOA, on 9 (2), 9 (1), 10 (1), 9 (2), 10 (2), 11 (1), 7 (3), 11 (1), and 10 (0) benchmark test functions. Thus, MSAO-EDA significantly outperforms the comparison algorithm on 20 D.

Summarizing the above analysis, we can conclude that the performance of MSAO-EDA on the CEC2022 test suite is significantly superior to SAO, EO, RIME, AFDBARO, CSOAOA, MRFO, EOSMA, JADE, and CFOA.

#### 4.5.2. Analysis for the Friedman Test Results

**Figure 12:**
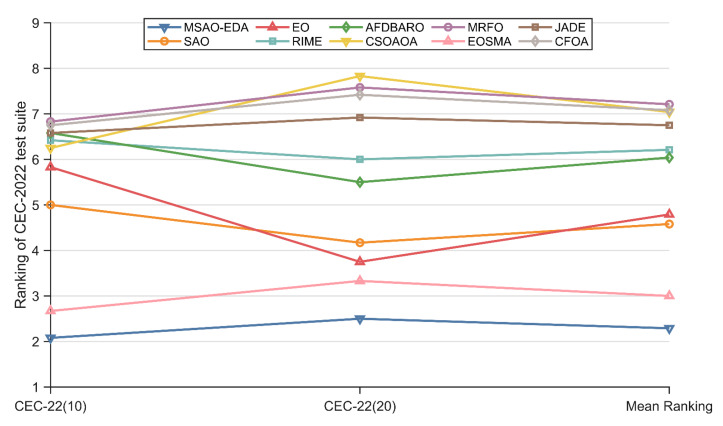
The Friedman test results of MSAO-EDA and its competitors on CEC2022.

In this section, the Friedman test is implemented to analyze the performance of MSAO-EDA and the competitors on the CEC2022 test suite with different dimensions. Table 10 summarizes the Friedman scores of these algorithms, which are visualized in Figure 12. According to Table 10, the Friedman *p*-values in the last column are all less than 0.05, which indicates there is a significant difference between MSAO-EDA and competitors. The specific analysis is as follows.

For 10 D, MSAO-EDA ranks first, followed by EOSMA, SAO, EO, CSOAOA, RIME, AFDBARO, JADE, CFOA, and MRFO.

For 20 D, MSAO-EDA ranks first, followed by EOSMA, EO, SAO, AFDBARO, RIME, JADE, CFOA, MRFO, and CSOAOA.

In conclusion, the overall performance of MSAO-EDA on the CEC2022 test suite is the best and outperforms the basic SAO in all cases. Therefore, the results of the Friedman test support the fact that MSAO-EDA is a high-performance method.

#### 4.5.3. Analysis for the Convergence Ability and Stability

**Figure A7:**
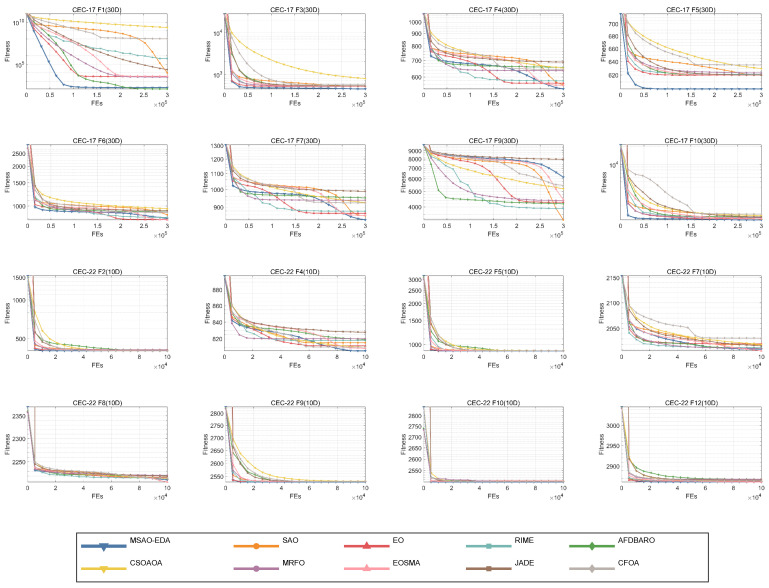
The convergence graphs of MSAO-EDA and its competitors on CEC2022 (10 D).

**Figure A8:**
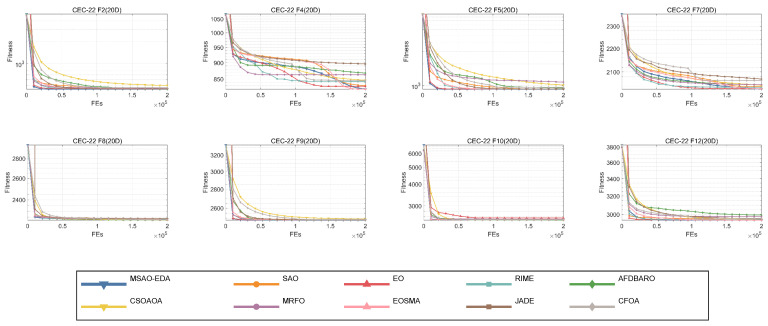
The convergence graphs of MSAO-EDA and its competitors on CEC2022 (20 D).

**Figure A9:**
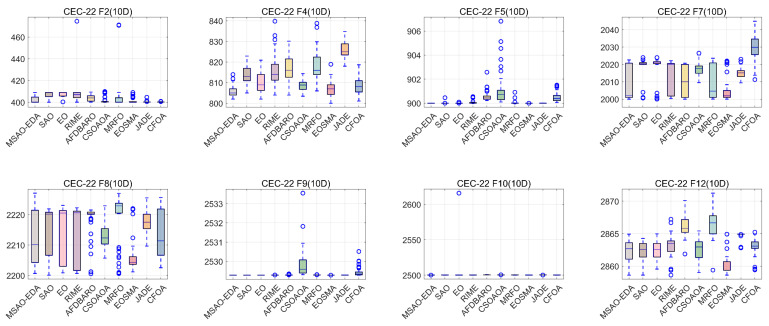
The box graphs of MSAO-EDA and its competitors on CEC2022 (10 D).

**Figure A10:**
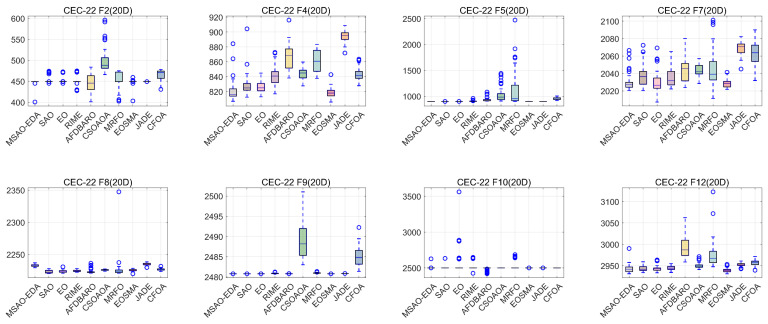
The box graphs of MSAO-EDA and its competitors on CEC2022 (20 D).

In this section, the convergence and stability of MSAO-EDA on low-dimensional functions are further analyzed by showing its results on the CEC2022 test suite. Also, for convenience, one each of the four types of functions (F1, F3, F6, and F11) are selected for this section. The complete convergence and box plots are shown in Figure A7, Figure A8, Figure A9 and Figure A10 in Appendix B.

**Figure 13:**
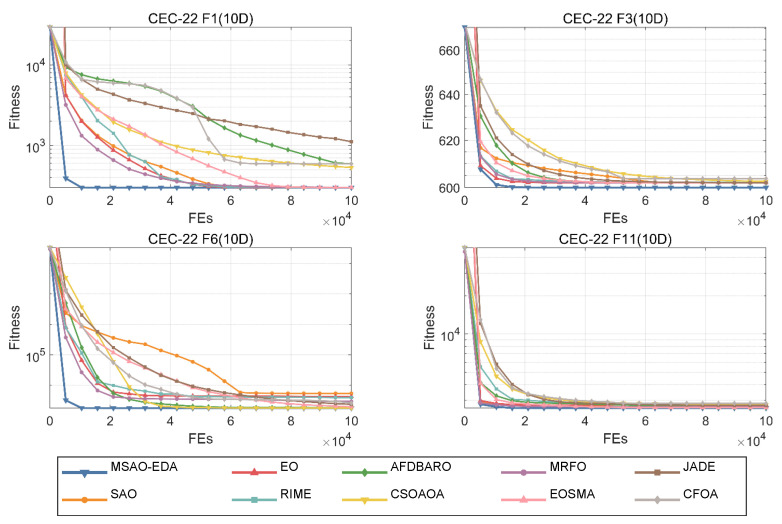
The convergence graphs of MSAO-EDA and its competitors on CEC2022 10 D.

Figure 13 shows the convergence curves of MSAO-EDA and the competitors for the four functions under CEC2022 10 D. In terms of convergence speed, MSAO-EDA shows the fastest speed and maintains higher accuracy. Therefore, MSAO-EDA has the same great convergence ability for low-dimensional functions.

**Figure 14:**
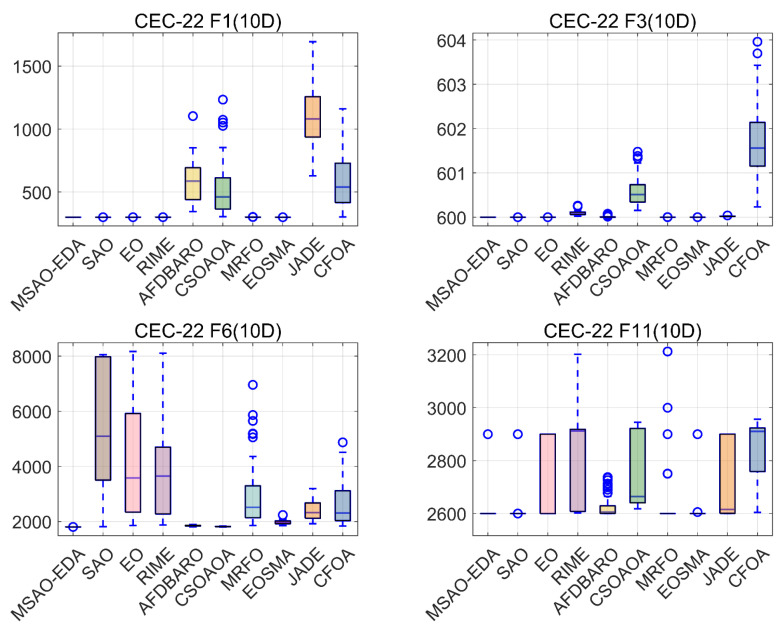
The box graphs of MSAO-EDA and its competitors on CEC2022 10 D.

The box plots of the results obtained by MSAO-EDA and the competitors when solving the CEC2022 10 D functions are shown in Figure 14. From Figure 14, MSAO-EDA provides a more centralized solution and a smaller overall distribution, demonstrating consistently superior performance.

## 5. Conclusions

In this paper, we propose a novel snow ablation algorithm variant, MSAO-EDA, which employs a new collaborative search framework to hybridize SAO and distribution estimation algorithms. Moreover, an offset EDA method is incorporated into the exploitation phase of SAO. The superior performance of MSAO-EDA is verified by comparing it with different types of basic or improved algorithms. Test results on the CEC2017 test suite (10 D/30 D/50 D/100 D) and the CEC2022 test suite (10 D/20 D) show that MSAO-EDA outperforms the competitors involved in the experiments, including four basic algorithms (EO, RIME, MRFO, and CFOA) and four improved algorithms (AFDBARO, CSOAOA, EOSMA, and JADE). The Wilcoxon rank sum test, Friedman test, convergence analysis, and robustness analysis confirm that the MSAO-EDA proposed in this paper is an excellent SAO variant. In our future work, we will focus on two directions. First, we will further enhance the collaborative search framework and try to extend it to other metaheuristic algorithms. Second, we will apply MSAO-EDA to tackle some real-world optimization problems, while we will also develop binary and multi-objective versions to solve more types of optimization problems.

## Figures and Tables

**Table 1:** CEC 2017 test functions.

Type	ID	CEC 2017 Function Name	Search Range	f_min_
Unimodal	F1	Shifted and Rotated Bent Cigar Function	[−100,100]	100
	F2	Shifted and Rotated Zakharov Function	[−100,100]	300
Multimodal	F3	Shifted and Rotated Rosenbrock’s Function	[−100,100]	400
	F4	Shifted and Rotated Rastrigin’s Function	[−100,100]	500
	F5	Shifted and Rotated Expanded Scaffer’s F6 Function	[−100,100]	600
	F6	Shifted and Rotated Lunacek Bi_Rastrigin Function	[−100,100]	700
	F7	Shifted and Rotated Non-Continuous Rastrigin’s Function	[−100,100]	800
	F8	Shifted and Rotated Levy Function	[−100,100]	900
	F9	Shifted and Rotated Schwefel’s Function	[−100,100]	1000
Hybrid	F10	Hybrid Function 1 (*N* = 3)	[−100,100]	1100
	F11	Hybrid Function 2 (*N* = 3)	[−100,100]	1200
	F12	Hybrid Function 3 (*N* = 3)	[−100,100]	1300
	F13	Hybrid Function 4 (*N* = 4)	[−100,100]	1400
	F14	Hybrid Function 5 (*N* = 4)	[−100,100]	1500
	F15	Hybrid Function 6 (*N* = 4)	[−100,100]	1600
	F16	Hybrid Function 6 (*N* = 5)	[−100,100]	1700
	F17	Hybrid Function 6 (*N* = 5)	[−100,100]	1800
	F18	Hybrid Function 6 (*N* = 5)	[−100,100]	1900
	F19	Hybrid Function 6 (*N* = 6)	[−100,100]	2000
Composition	F20	Composition Function 1 (*N* = 3)	[−100,100]	2100
	F21	Composition Function 2 (*N* = 3)	[−100,100]	2200
	F22	Composition Function 3 (*N* = 4)	[−100,100]	2300
	F23	Composition Function 4 (*N* = 4)	[−100,100]	2400
	F24	Composition Function 5 (*N* = 5)	[−100,100]	2500
	F25	Composition Function 6 (*N* = 5)	[−100,100]	2600
	F26	Composition Function 7 (*N* = 6)	[−100,100]	2700
	F27	Composition Function 8 (*N* = 6)	[−100,100]	2800
	F28	Composition Function 9 (*N* = 3)	[−100,100]	2900
	F29	Composition Function 10 (*N* = 3)	[−100,100]	3000

**Table 2:** CEC 2022 test functions.

Type	ID	CEC 2022 Function Name	Search Range	f_min_
Unimodal functions	F1	Shifted and full Rotated Zakharov Function	[−100,100]	300
Multimodal functions	F2	Shifted and full Rotated Rosenbrock’s Function	[−100,100]	400
	F3	Shifted and full Rotated Expanded Schaffer’s f6 Function	[−100,100]	600
	F4	Shifted and full Rotated Non-Continuous Rastrigin’s Function	[−100,100]	800
	F5	Shifted and full Rotated Levy Function	[−100,100]	900
Hybrid functions	F6	Hybrid Function 1 (*N* = 3)	[−100,100]	1800
	F7	Hybrid Function 2 (*N* = 6)	[−100,100]	2000
	F8	Hybrid Function 3 (*N* = 5)	[−100,100]	2200
Composition functions	F9	Composition Function 1 (*N* = 5)	[−100,100]	2300
	F10	Composition Function 2 (*N* = 4)	[−100,100]	2400
	F11	Composition Function 3 (*N* = 5)	[−100,100]	2600
	F12	Composition Function 4 (*N* = 6)	[−100,100]	2700

**Table 3:** The parameter settings for ten algorithms.

Algorithm	Parameter Values
MSAO-EDA	$M\in \left(0.35,\;0.6\right),\rho =0.8,\;N_{d}=0.35N$
SAO	$M\in \left(0.35,\;0.6\right)$
EO	$a_{1}=2,\;a_{2}=1\;,GP=0.5$
RIME	W=5
MRFO	S=2
CFOA	$per\in \left(3\;,4\right)$
AFDBARO	k=1
CSOAOA	$Mop_{\max}=1,\;Mop_{\min}=0.2\;,\alpha =5\;,\mu =0.499$
EOSMA	$a_{1}=2\;,a_{2}=1\;,GP=0.5\;,z=0.6\;,q=0.2$
JADE	c=0.1 ,p=0.05 ,μCR=0.5 ,μF=0.5

**Table 4:** Influence of the parameter *ρ* on the performance of MSAO-EDA.

Algorithm		SAO	ρ = 0.1	ρ = 0.2	ρ = 0.3	ρ = 0.4	ρ = 0.5	ρ = 0.6	ρ = 0.7	ρ = 0.8	ρ = 0.9	ρ = 1.0	Friedman-*p*-Value
CEC-2017 Dim	10	7.90	6.95	6.53	6.59	6.38	4.97	5.52	4.83	5.95	4.91	5.48	4.38E-03
	30	7.79	7.24	6.55	6.03	5.72	6.07	5.62	5.52	4.52	5.17	5.76	1.36E-02
	50	8.72	7.79	6.90	5.69	5.38	5.72	4.90	4.90	5.28	5.34	5.38	8.91E-06
	100	9.59	9.17	8.07	7.72	6.62	4.90	4.72	3.93	3.93	3.52	3.83	5.75E-25
Mean rank		8.50	7.79	7.01	6.51	6.03	5.41	5.19	4.79	4.92	4.74	5.11	N/A
CEC-2022 Dim	10	8.67	7.58	7.29	5.96	4.96	6.92	3.54	5.67	4.75	5.92	4.75	1.17E-03
	20	7.08	6.42	6.08	6.25	7.58	4.50	6.33	6.25	5.25	5.46	4.79	4.90E-01
Mean rank		20	7.00	6.69	6.10	6.27	5.71	4.94	5.96	5.00	5.69	4.77	2.45E-01
Total mean rank		8.29	7.53	6.90	6.37	6.11	5.51	5.11	5.18	4.95	5.05	5.00	N/A

**Table 5:** Details of MSAO-EDA and its variants.

Strategy	MSAO-EDA-1	MSAO-EDA-2	MSAO-EDA
Collaborative search framework	Yes	No	Yes
Offset EDA strategy	No	Yes	Yes

**Table 6:** Friedman results of MSAO-EDA and its variants.

Algorithm	CEC-2017 Test Suite				CEC-2022 Test Suite		Mean Rank of CEC-2017	Mean Rank of CEC-2022	Total Mean Rank of Total
	Dim				Dim				
	10	30	50	100	10	20			
SAO	7.90	7.79	8.72	9.59	8.67	7.08	8.50	7.88	8.29
MSAO-EDA-1	6.95	7.24	7.79	9.17	7.58	6.42	7.79	7.00	7.53
MSAO-EDA-2	6.53	6.55	6.90	8.07	7.29	6.08	7.01	6.69	6.90
MSAO-EDA	6.59	6.03	5.69	7.72	5.96	6.25	6.51	6.10	6.37
Friedman-*p*-value	1.72E-06	3.13E-11	1.69E-07	3.57E-13	1.72E-06	3.63E-03	N/A	N/A	N/A

**Table 7:** Wilcoxon rank sum results of MSAO-EDA and its competitors on CEC2017.

MSAO-EDA vs. +/=/−	CEC-2017 Test Suite			
	10 D	30 D	50 D	100 D
SAO	23/3/3	26/2/1	24/4/1	27/1/1
EO	23/2/4	25/2/2	20/6/3	18/6/5
RIME	24/2/3	27/1/1	25/2/2	24/2/3
AFDBARO	23/2/4	26/0/3	25/1/3	23/2/4
CSOAOA	21/3/5	27/0/2	25/2/2	25/1/3
MRFO	23/1/5	28/0/1	26/1/2	24/2/3
EOSMA	13/7/9	25/2/2	18/6/5	19/3/7
JADE	24/2/3	29/0/0	28/0/1	27/1/1
CFOA	22/1/6	27/1/1	27/0/2	25/1/3

**Table 8:** Friedman results of MSAO-EDA and its competitors on CEC2017.

Algorithm	CEC-2017 Test Suite				
	10 D	30 D	50 D	100 D	Average Ranking
MSAO-EDA	2.98	1.59	2.31	2.45	2.33
SAO	6.14	4.48	6.97	8.17	6.44
EO	6.22	4.07	3.21	2.90	4.10
RIME	6.76	6.55	5.83	5.45	6.15
AFDBARO	5.14	6.28	6.17	5.24	5.71
CSOAOA	4.66	7.14	7.31	8.52	6.91
MRFO	6.72	6.17	6.34	5.41	6.16
EOSMA	2.76	3.00	2.38	2.76	2.72
JADE	7.03	8.07	7.34	6.41	7.22
CFOA	6.59	7.66	7.14	7.69	7.27
Friedman-*p*-value	1.28E-11	2.02E-23	1.29E-21	1.45E-26	N/A

**Table 9:** Wilcoxon rank sum results of MSAO-EDA and its competitors on CEC2022.

MSAO-EDA vs. +/=/−	CEC-2022 Test Suite	
	10 D	20 D
SAO	9/3/0	9/1/2
EO	8/4/0	9/2/1
RIME	9/3/0	10/1/1
AFDBARO	11/1/0	9/1/2
CSOAOA	8/4/0	10/0/2
MRFO	10/2/0	11/0/1
EOSMA	7/3/2	7/2/3
JADE	10/2/0	11/0/1
CFOA	10/2/0	10/0/2

**Table 10:** Friedman test results of MSAO-EDA and its competitors on CEC2022.

Algorithm	CEC-2022 Test Suite		
	10 D	20 D	Average Ranking
MSAO-EDA	2.08	2.50	2.29
SAO	5.00	4.17	4.58
EO	5.83	3.75	4.79
RIME	6.42	6.00	6.21
AFDBARO	6.58	5.50	6.04
CSOAOA	6.25	7.83	7.04
MRFO	6.83	7.58	7.21
EOSMA	2.67	3.33	3.00
JADE	6.58	6.92	6.75
CFOA	6.75	7.42	7.08
Friedman-*p*-value	4.63E-05	9.89E-07	N/A

## Data Availability

The data are provided within the manuscript.

## Appendix A

**Table A1:** Comparison results on of MSAO-EDA and its competitors on CEC2017 (10 D).

No.	Metric	MSAO-EDA	SAO	EO	RIME	AFDBARO	CSOAOA	MRFO	EOSMA	JADE	CFOA
F1	Best	1.000E+02	1.002E+02	1.001E+02	6.126E+02	1.009E+02	3.786E+04	1.000E+02	1.008E+02	8.084E+02	2.017E+02
	Mean	1.000E+02	2.854E+03	2.585E+03	9.754E+03	1.119E+02	8.466E+06	2.358E+03	1.374E+03	1.063E+04	1.380E+04
	Std	0.000E+00	3.119E+03	2.817E+03	7.491E+03	1.627E+01	1.709E+07	2.599E+03	1.565E+03	4.729E+03	2.514E+04
	Rank	1	6	5	7	2	10	4	3	8	9
F2	Best	3.000E+02	3.000E+02	3.000E+02	3.000E+02	3.327E+02	3.067E+02	3.000E+02	3.000E+02	7.391E+02	3.037E+02
	Mean	3.000E+02	3.000E+02	3.000E+02	3.002E+02	4.724E+02	5.947E+02	3.000E+02	3.000E+02	1.394E+03	6.684E+02
	Std	0.000E+00	6.043E-11	4.250E-07	1.328E-01	1.085E+02	2.847E+02	3.695E-04	6.677E-04	3.253E+02	4.739E+02
	Rank	1	2	3	6	7	8	4	5	10	9
F3	Best	4.007E+02	4.027E+02	4.038E+02	4.010E+02	4.002E+02	4.003E+02	4.000E+02	4.001E+02	4.036E+02	4.006E+02
	Mean	4.022E+02	4.037E+02	4.046E+02	4.056E+02	4.034E+02	4.035E+02	4.019E+02	4.014E+02	4.043E+02	4.034E+02
	Std	6.438E-01	3.646E-01	3.274E-01	1.445E+00	1.952E+00	1.783E+00	1.339E+00	4.985E-01	2.465E-01	1.370E+00
	Rank	3	7	9	10	4	6	2	1	8	5
F4	Best	5.010E+02	5.010E+02	5.030E+02	5.020E+02	5.062E+02	5.061E+02	5.040E+02	5.020E+02	5.106E+02	5.051E+02
	Mean	5.062E+02	5.086E+02	5.090E+02	5.101E+02	5.178E+02	5.121E+02	5.142E+02	5.063E+02	5.204E+02	5.121E+02
	Std	2.774E+00	3.893E+00	3.227E+00	3.736E+00	5.871E+00	3.004E+00	7.178E+00	2.401E+00	3.343E+00	3.371E+00
	Rank	1	3	4	5	9	7	8	2	10	6
F5	Best	6.000E+02	6.000E+02	6.000E+02	6.000E+02	6.000E+02	6.002E+02	6.000E+02	6.000E+02	6.000E+02	6.006E+02
	Mean	6.000E+02	6.000E+02	6.000E+02	6.001E+02	6.000E+02	6.006E+02	6.000E+02	6.000E+02	6.000E+02	6.018E+02
	Std	0.000E+00	2.173E-07	1.860E-06	4.629E-02	1.325E-02	3.223E-01	4.155E-04	3.598E-04	3.957E-03	8.930E-01
	Rank	1	2	3	8	6	9	4	5	7	10
F6	Best	7.111E+02	7.122E+02	7.118E+02	7.037E+02	7.112E+02	7.147E+02	7.150E+02	7.115E+02	7.215E+02	7.125E+02
	Mean	7.165E+02	7.175E+02	7.176E+02	7.201E+02	7.287E+02	7.246E+02	7.293E+02	7.163E+02	7.323E+02	7.188E+02
	Std	4.877E+00	3.893E+00	3.785E+00	5.283E+00	7.928E+00	2.958E+00	9.346E+00	2.581E+00	3.553E+00	3.223E+00
	Rank	2	3	4	6	8	7	9	1	10	5
F7	Best	8.020E+02	8.050E+02	8.030E+02	8.040E+02	8.032E+02	8.046E+02	8.050E+02	8.020E+02	8.132E+02	8.030E+02
	Mean	8.068E+02	8.112E+02	8.089E+02	8.099E+02	8.163E+02	8.093E+02	8.166E+02	8.065E+02	8.210E+02	8.091E+02
	Std	3.334E+00	4.360E+00	3.762E+00	3.626E+00	5.448E+00	2.169E+00	5.769E+00	2.227E+00	3.253E+00	3.316E+00
	Rank	2	7	3	6	8	5	9	1	10	4
F8	Best	9.000E+02	9.000E+02	9.000E+02	9.000E+02	9.002E+02	9.001E+02	9.000E+02	9.000E+02	9.000E+02	9.001E+02
	Mean	9.000E+02	9.000E+02	9.000E+02	9.000E+02	9.006E+02	9.009E+02	9.001E+02	9.000E+02	9.000E+02	9.007E+02
	Std	0.000E+00	6.362E-02	1.029E-13	7.359E-02	2.100E-01	1.038E+00	4.020E-01	4.973E-12	9.100E-05	4.992E-01
	Rank	1	5	1	6	8	10	7	3	4	9
F9	Best	1.015E+03	1.010E+03	1.004E+03	1.004E+03	1.066E+03	1.130E+03	1.000E+03	1.004E+03	1.880E+03	1.020E+03
	Mean	1.393E+03	1.344E+03	1.405E+03	1.284E+03	1.487E+03	1.432E+03	1.479E+03	1.258E+03	2.101E+03	1.610E+03
	Std	2.813E+02	1.859E+02	2.419E+02	1.578E+02	1.705E+02	1.297E+02	2.091E+02	1.859E+02	1.229E+02	2.256E+02
	Rank	4	3	5	2	8	6	7	1	10	9
F10	Best	1.100E+03	1.100E+03	1.100E+03	1.100E+03	1.104E+03	1.102E+03	1.100E+03	1.100E+03	1.104E+03	1.105E+03
	Mean	1.101E+03	1.105E+03	1.103E+03	1.109E+03	1.110E+03	1.107E+03	1.105E+03	1.103E+03	1.106E+03	1.116E+03
	Std	5.823E-01	3.420E+00	2.179E+00	4.288E+00	3.761E+00	2.393E+00	2.877E+00	1.403E+00	7.488E-01	6.534E+00
	Rank	1	4	3	8	9	7	5	2	6	10
F11	Best	1.201E+03	1.487E+03	2.691E+03	4.936E+03	2.659E+03	2.330E+03	1.286E+03	3.220E+03	4.009E+04	7.616E+03
	Mean	1.254E+03	1.106E+04	1.158E+04	5.346E+04	8.671E+03	7.108E+03	2.073E+04	8.190E+03	1.233E+05	2.785E+04
	Std	6.058E+01	7.196E+03	8.536E+03	4.704E+04	8.037E+03	3.908E+03	2.952E+04	5.161E+03	5.658E+04	2.448E+04
	Rank	1	5	6	9	4	2	7	3	10	8
F12	Best	1.300E+03	1.336E+03	1.338E+03	1.329E+03	1.307E+03	1.304E+03	1.313E+03	1.306E+03	1.317E+03	1.676E+03
	Mean	1.306E+03	7.114E+03	4.819E+03	1.045E+04	1.319E+03	1.316E+03	1.940E+03	1.330E+03	1.345E+03	2.218E+03
	Std	3.171E+00	5.943E+03	3.352E+03	8.291E+03	5.626E+00	8.084E+00	5.247E+02	1.174E+01	1.104E+01	2.861E+02
	Rank	1	9	8	10	3	2	6	4	5	7
F13	Best	1.400E+03	1.402E+03	1.407E+03	1.404E+03	1.401E+03	1.403E+03	1.431E+03	1.403E+03	1.411E+03	1.420E+03
	Mean	1.406E+03	1.422E+03	1.439E+03	1.427E+03	1.408E+03	1.407E+03	1.457E+03	1.415E+03	1.418E+03	1.445E+03
	Std	6.223E+00	1.721E+01	1.894E+01	1.588E+01	3.928E+00	2.936E+00	1.985E+01	8.652E+00	3.017E+00	1.362E+01
	Rank	1	6	8	7	3	2	10	4	5	9
F14	Best	1.500E+03	1.501E+03	1.508E+03	1.503E+03	1.502E+03	1.501E+03	1.534E+03	1.501E+03	1.503E+03	1.515E+03
	Mean	1.500E+03	1.546E+03	1.540E+03	1.527E+03	1.505E+03	1.503E+03	1.620E+03	1.507E+03	1.504E+03	1.615E+03
	Std	2.349E-01	7.728E+01	3.760E+01	4.784E+01	1.802E+00	1.147E+00	7.640E+01	3.153E+00	8.475E-01	7.891E+01
	Rank	1	8	7	6	4	2	10	5	3	9
F15	Best	1.600E+03	1.600E+03	1.600E+03	1.601E+03	1.600E+03	1.602E+03	1.601E+03	1.601E+03	1.604E+03	1.602E+03
	Mean	1.604E+03	1.633E+03	1.620E+03	1.639E+03	1.651E+03	1.607E+03	1.645E+03	1.602E+03	1.612E+03	1.620E+03
	Std	6.741E+00	4.218E+01	2.240E+01	5.329E+01	5.924E+01	3.585E+00	7.750E+01	1.141E+00	4.168E+00	2.135E+01
	Rank	2	7	6	8	10	3	9	1	4	5
F16	Best	1.700E+03	1.701E+03	1.700E+03	1.701E+03	1.701E+03	1.713E+03	1.701E+03	1.701E+03	1.723E+03	1.723E+03
	Mean	1.713E+03	1.720E+03	1.723E+03	1.718E+03	1.714E+03	1.723E+03	1.727E+03	1.718E+03	1.731E+03	1.736E+03
	Std	1.281E+01	1.231E+01	1.411E+01	1.328E+01	1.023E+01	5.385E+00	1.836E+01	9.342E+00	3.140E+00	8.636E+00
	Rank	1	5	7	3	2	6	8	4	9	10
F17	Best	1.800E+03	3.469E+03	1.913E+03	1.868E+03	1.813E+03	1.803E+03	1.951E+03	1.829E+03	1.835E+03	2.030E+03
	Mean	1.804E+03	1.759E+04	1.529E+04	9.141E+03	1.823E+03	1.811E+03	4.508E+03	1.863E+03	1.853E+03	2.731E+03
	Std	7.382E+00	9.357E+03	1.321E+04	8.371E+03	3.723E+00	6.090E+00	2.566E+03	1.596E+01	9.105E+00	3.867E+02
	Rank	1	10	9	8	3	2	7	5	4	6
F18	Best	1.900E+03	1.902E+03	1.906E+03	1.902E+03	1.901E+03	1.901E+03	1.910E+03	1.903E+03	1.902E+03	1.910E+03
	Mean	1.900E+03	2.011E+03	1.931E+03	1.910E+03	1.902E+03	1.901E+03	1.982E+03	1.906E+03	1.903E+03	1.953E+03
	Std	3.942E-01	4.526E+02	2.447E+01	9.526E+00	8.403E-01	5.227E-01	6.972E+01	1.377E+00	4.815E-01	3.420E+01
	Rank	1	10	7	6	3	2	9	5	4	8
F19	Best	2.000E+03	2.000E+03	2.000E+03	2.000E+03	2.000E+03	2.009E+03	2.000E+03	2.000E+03	2.011E+03	2.010E+03
	Mean	2.004E+03	2.014E+03	2.013E+03	2.011E+03	2.007E+03	2.028E+03	2.009E+03	2.009E+03	2.020E+03	2.042E+03
	Std	7.217E+00	1.279E+01	1.102E+01	9.352E+00	7.036E+00	6.646E+00	1.037E+01	9.323E+00	3.885E+00	1.358E+01
	Rank	1	7	6	5	2	9	3	4	8	10
F20	Best	2.200E+03	2.200E+03	2.200E+03	2.100E+03	2.201E+03	2.104E+03	2.101E+03	2.200E+03	2.204E+03	2.201E+03
	Mean	2.240E+03	2.217E+03	2.250E+03	2.217E+03	2.202E+03	2.201E+03	2.208E+03	2.202E+03	2.226E+03	2.203E+03
	Std	5.260E+01	3.983E+01	5.547E+01	4.411E+01	7.138E-01	1.395E+01	3.395E+01	1.506E+01	3.448E+01	2.727E+00
	Rank	9	7	10	6	3	1	5	2	8	4
F21	Best	2.200E+03	2.200E+03	2.200E+03	2.200E+03	2.201E+03	2.200E+03	2.200E+03	2.200E+03	2.301E+03	2.225E+03
	Mean	2.298E+03	2.293E+03	2.294E+03	2.291E+03	2.249E+03	2.255E+03	2.288E+03	2.269E+03	2.302E+03	2.293E+03
	Std	1.401E+01	2.505E+01	2.377E+01	3.098E+01	3.349E+01	4.421E+01	3.263E+01	3.966E+01	4.732E-01	2.412E+01
	Rank	9	6	8	5	1	2	4	3	10	7
F22	Best	2.600E+03	2.603E+03	2.604E+03	2.606E+03	2.300E+03	2.602E+03	2.300E+03	2.600E+03	2.611E+03	2.314E+03
	Mean	2.606E+03	2.612E+03	2.612E+03	2.613E+03	2.617E+03	2.609E+03	2.610E+03	2.607E+03	2.619E+03	2.600E+03
	Std	3.360E+00	4.059E+00	4.002E+00	4.035E+00	4.586E+01	3.048E+00	4.497E+01	2.614E+00	3.495E+00	5.817E+01
	Rank	2	7	6	8	9	4	5	3	10	1
F23	Best	2.500E+03	2.500E+03	2.500E+03	2.500E+03	2.451E+03	2.501E+03	2.500E+03	2.400E+03	2.526E+03	2.505E+03
	Mean	2.709E+03	2.670E+03	2.708E+03	2.684E+03	2.508E+03	2.509E+03	2.687E+03	2.587E+03	2.655E+03	2.544E+03
	Std	6.978E+01	1.107E+02	8.398E+01	1.086E+02	2.933E+01	3.293E+01	1.052E+02	1.162E+02	7.979E+01	4.746E+01
	Rank	10	6	9	7	1	2	8	4	5	3
F24	Best	2.898E+03	2.898E+03	2.898E+03	2.898E+03	2.600E+03	2.898E+03	2.898E+03	2.898E+03	2.898E+03	2.898E+03
	Mean	2.920E+03	2.928E+03	2.929E+03	2.923E+03	2.844E+03	2.905E+03	2.920E+03	2.898E+03	2.914E+03	2.899E+03
	Std	2.298E+01	2.265E+01	2.234E+01	2.388E+01	1.140E+02	1.352E+01	2.293E+01	6.838E-01	2.071E+01	8.337E-01
	Rank	7	9	10	8	1	4	6	2	5	3
F25	Best	2.900E+03	2.900E+03	2.900E+03	2.803E+03	2.601E+03	2.623E+03	2.800E+03	2.600E+03	2.900E+03	2.653E+03
	Mean	2.900E+03	2.903E+03	2.924E+03	2.910E+03	2.805E+03	2.858E+03	2.898E+03	2.894E+03	2.900E+03	2.897E+03
	Std	0.000E+00	1.179E+01	3.288E+01	2.928E+01	1.190E+02	1.022E+02	1.400E+01	4.196E+01	7.063E-04	3.497E+01
	Rank	6	8	10	9	1	2	5	3	7	4
F26	Best	3.089E+03	3.089E+03	3.089E+03	3.088E+03	3.090E+03	3.090E+03	3.089E+03	3.087E+03	3.094E+03	3.089E+03
	Mean	3.091E+03	3.090E+03	3.090E+03	3.092E+03	3.096E+03	3.091E+03	3.098E+03	3.089E+03	3.094E+03	3.092E+03
	Std	1.940E+00	1.541E+00	6.906E-01	2.454E+00	2.854E+00	1.269E+00	1.185E+01	4.707E-01	1.905E-01	1.930E+00
	Rank	4	3	2	7	9	5	10	1	8	6
F27	Best	3.100E+03	3.100E+03	3.100E+03	3.100E+03	2.848E+03	2.924E+03	2.800E+03	2.805E+03	3.100E+03	2.959E+03
	Mean	3.117E+03	3.217E+03	3.212E+03	3.167E+03	3.123E+03	3.105E+03	3.144E+03	3.094E+03	3.100E+03	3.122E+03
	Std	6.743E+01	1.411E+02	1.474E+02	8.959E+01	5.382E+01	3.028E+01	1.337E+02	4.128E+01	4.231E-01	3.765E+01
	Rank	4	10	9	8	6	3	7	1	2	5
F28	Best	3.129E+03	3.130E+03	3.131E+03	3.132E+03	3.140E+03	3.143E+03	3.144E+03	3.132E+03	3.164E+03	3.139E+03
	Mean	3.152E+03	3.155E+03	3.149E+03	3.164E+03	3.179E+03	3.163E+03	3.187E+03	3.142E+03	3.191E+03	3.168E+03
	Std	9.849E+00	1.989E+01	1.179E+01	2.295E+01	1.882E+01	1.093E+01	3.463E+01	5.657E+00	1.084E+01	1.450E+01
	Rank	3	4	2	6	8	5	9	1	10	7
F29	Best	3.395E+03	3.535E+03	3.699E+03	4.122E+03	4.798E+03	3.734E+03	3.854E+03	3.605E+03	5.752E+03	3.658E+03
	Mean	4.415E+04	2.699E+05	2.712E+05	6.121E+04	9.535E+04	8.647E+03	2.397E+05	4.577E+03	2.565E+04	1.466E+04
	Std	2.070E+05	3.992E+05	3.980E+05	1.924E+05	1.946E+05	1.215E+04	5.050E+05	6.836E+02	1.114E+04	1.956E+04
	Rank	5	9	10	6	7	2	8	1	4	3

**Table A2:** Comparison results on of MSAO-EDA and its competitors on CEC2017 (30 D).

No.	Metric	MSAO-EDA	SAO	EO	RIME	AFDBARO	CSOAOA	MRFO	EOSMA	JADE	CFOA
F1	Best	1.000E+02	2.478E+03	1.070E+02	1.198E+05	1.006E+02	7.267E+08	1.008E+02	1.003E+02	1.075E+04	3.222E+07
	Mean	1.779E+02	1.001E+04	3.438E+03	4.932E+05	1.048E+02	2.264E+09	3.153E+03	4.016E+03	2.419E+04	1.069E+08
	Std	5.345E+02	5.401E+03	4.068E+03	2.948E+05	6.561E+00	9.431E+08	3.807E+03	4.799E+03	9.062E+03	5.444E+07
	Rank	2	6	4	8	1	10	3	5	7	9
F2	Best	3.000E+02	7.930E+03	3.618E+02	4.345E+02	1.385E+04	1.614E+04	5.479E+02	1.126E+03	3.834E+04	6.350E+03
	Mean	3.000E+02	1.579E+04	5.329E+02	7.048E+02	3.839E+04	2.617E+04	1.403E+03	2.186E+03	5.624E+04	1.675E+04
	Std	9.423E-12	5.072E+03	1.692E+02	1.595E+02	1.184E+04	4.726E+03	5.489E+02	6.106E+02	7.072E+03	4.882E+03
	Rank	1	6	2	3	9	8	4	5	10	7
F3	Best	4.000E+02	4.721E+02	4.679E+02	4.207E+02	4.512E+02	5.852E+02	4.001E+02	4.451E+02	4.870E+02	4.950E+02
	Mean	4.458E+02	4.958E+02	4.938E+02	4.970E+02	4.888E+02	7.730E+02	4.896E+02	4.916E+02	5.063E+02	5.380E+02
	Std	3.143E+01	1.235E+01	1.604E+01	2.057E+01	1.781E+01	1.811E+02	2.860E+01	1.814E+01	1.242E+01	1.831E+01
	Rank	1	6	5	7	2	10	3	4	8	9
F4	Best	5.099E+02	5.187E+02	5.219E+02	5.311E+02	5.802E+02	5.954E+02	5.448E+02	5.159E+02	6.388E+02	5.953E+02
	Mean	5.370E+02	5.380E+02	5.470E+02	5.595E+02	6.347E+02	6.315E+02	6.162E+02	5.354E+02	6.693E+02	6.243E+02
	Std	2.742E+01	1.143E+01	1.399E+01	1.784E+01	2.185E+01	1.616E+01	3.647E+01	1.038E+01	8.972E+00	1.531E+01
	Rank	2	3	4	5	9	8	6	1	10	7
F5	Best	6.000E+02	6.000E+02	6.000E+02	6.003E+02	6.000E+02	6.060E+02	6.000E+02	6.000E+02	6.001E+02	6.098E+02
	Mean	6.000E+02	6.001E+02	6.000E+02	6.010E+02	6.001E+02	6.097E+02	6.032E+02	6.000E+02	6.002E+02	6.152E+02
	Std	7.570E-08	3.418E-02	1.234E-03	5.131E-01	1.736E-01	2.012E+00	5.695E+00	1.400E-02	1.284E-02	2.608E+00
	Rank	1	4	2	7	5	9	8	3	6	10
F6	Best	7.439E+02	7.536E+02	7.526E+02	7.674E+02	8.217E+02	8.814E+02	7.842E+02	7.485E+02	8.635E+02	8.228E+02
	Mean	8.054E+02	8.401E+02	7.714E+02	8.012E+02	8.823E+02	9.353E+02	8.787E+02	7.664E+02	9.058E+02	8.763E+02
	Std	5.637E+01	4.815E+01	1.250E+01	1.613E+01	3.144E+01	2.220E+01	5.356E+01	1.121E+01	1.028E+01	2.327E+01
	Rank	4	5	2	3	8	10	7	1	9	6
F7	Best	8.109E+02	8.170E+02	8.179E+02	8.274E+02	8.773E+02	8.801E+02	8.587E+02	8.229E+02	9.370E+02	8.637E+02
	Mean	8.343E+02	8.352E+02	8.479E+02	8.586E+02	9.315E+02	9.056E+02	9.187E+02	8.370E+02	9.697E+02	9.033E+02
	Std	2.612E+01	1.039E+01	1.450E+01	1.335E+01	2.221E+01	1.101E+01	2.716E+01	9.532E+00	8.866E+00	1.605E+01
	Rank	1	2	4	5	9	7	8	3	10	6
F8	Best	9.000E+02	9.000E+02	9.000E+02	9.028E+02	9.296E+02	1.005E+03	9.191E+02	9.001E+02	9.001E+02	1.113E+03
	Mean	9.000E+02	9.007E+02	9.009E+02	9.593E+02	1.513E+03	1.492E+03	2.099E+03	9.017E+02	9.002E+02	1.315E+03
	Std	0.000E+00	1.677E+00	9.578E-01	6.762E+01	5.524E+02	5.079E+02	1.091E+03	1.757E+00	4.592E-02	1.517E+02
	Rank	1	3	4	6	9	8	10	5	2	7
F9	Best	2.345E+03	1.765E+03	1.908E+03	2.445E+03	3.329E+03	4.523E+03	2.909E+03	3.168E+03	6.964E+03	3.977E+03
	Mean	6.151E+03	3.297E+03	4.262E+03	3.890E+03	4.166E+03	5.179E+03	4.367E+03	4.422E+03	7.925E+03	5.447E+03
	Std	1.879E+03	1.326E+03	7.983E+02	5.648E+02	4.076E+02	2.418E+02	5.786E+02	6.354E+02	3.275E+02	6.523E+02
	Rank	9	1	4	2	3	7	5	6	10	8
F10	Best	1.103E+03	1.111E+03	1.111E+03	1.157E+03	1.158E+03	1.204E+03	1.144E+03	1.111E+03	1.200E+03	1.277E+03
	Mean	1.121E+03	1.140E+03	1.154E+03	1.251E+03	1.237E+03	1.303E+03	1.202E+03	1.153E+03	1.237E+03	1.389E+03
	Std	2.375E+01	2.644E+01	3.103E+01	4.486E+01	3.439E+01	5.609E+01	3.839E+01	2.981E+01	1.648E+01	7.075E+01
	Rank	1	2	4	8	7	9	5	3	6	10
F11	Best	1.201E+03	1.053E+05	2.720E+04	3.390E+05	6.402E+04	3.868E+06	2.798E+04	1.503E+04	9.189E+05	4.932E+05
	Mean	1.450E+03	8.211E+05	1.074E+05	6.322E+06	8.256E+05	2.214E+07	2.714E+05	6.292E+04	2.111E+06	4.811E+06
	Std	1.866E+02	5.887E+05	1.145E+05	4.809E+06	5.323E+05	1.177E+07	2.345E+05	3.705E+04	4.913E+05	2.938E+06
	Rank	1	5	3	9	6	10	4	2	7	8
F12	Best	1.338E+03	1.578E+03	2.245E+03	1.393E+04	2.016E+03	9.479E+03	1.377E+03	1.424E+03	6.507E+04	1.493E+04
	Mean	1.365E+03	2.670E+04	2.466E+04	8.147E+04	7.456E+03	1.758E+05	1.664E+04	1.141E+04	1.633E+05	2.627E+04
	Std	1.828E+01	2.210E+04	2.003E+04	5.965E+04	1.141E+04	2.094E+05	1.737E+04	1.003E+04	4.352E+04	1.187E+04
	Rank	1	7	5	8	2	10	4	3	9	6
F13	Best	1.427E+03	2.078E+03	2.334E+03	2.806E+03	1.508E+03	1.469E+03	1.761E+03	1.537E+03	3.705E+03	1.922E+03
	Mean	1.452E+03	1.717E+04	7.685E+03	1.262E+04	2.034E+03	1.505E+03	4.495E+03	1.596E+03	9.446E+03	7.290E+03
	Std	1.001E+01	1.203E+04	5.448E+03	8.507E+03	9.574E+02	2.241E+01	3.881E+03	3.878E+01	3.143E+03	4.984E+03
	Rank	1	10	7	9	4	2	5	3	8	6
F14	Best	1.520E+03	1.563E+03	1.530E+03	4.083E+03	1.594E+03	1.922E+03	1.596E+03	1.678E+03	1.100E+04	2.467E+03
	Mean	1.530E+03	4.061E+03	5.361E+03	1.879E+04	2.373E+03	2.398E+03	9.906E+03	2.261E+03	3.148E+04	1.412E+04
	Std	5.067E+00	3.242E+03	4.659E+03	1.486E+04	3.898E+03	3.310E+02	8.492E+03	5.149E+02	9.931E+03	9.941E+03
	Rank	1	5	6	9	3	4	7	2	10	8
F15	Best	1.616E+03	1.618E+03	1.619E+03	1.663E+03	2.029E+03	2.161E+03	1.977E+03	1.621E+03	2.474E+03	2.059E+03
	Mean	2.062E+03	2.068E+03	2.171E+03	2.253E+03	2.541E+03	2.384E+03	2.468E+03	1.982E+03	2.924E+03	2.514E+03
	Std	3.397E+02	2.326E+02	2.758E+02	2.576E+02	2.474E+02	1.190E+02	2.318E+02	1.865E+02	1.538E+02	2.110E+02
	Rank	2	3	4	5	9	6	7	1	10	8
F16	Best	1.731E+03	1.738E+03	1.729E+03	1.757E+03	1.766E+03	1.778E+03	1.760E+03	1.739E+03	1.910E+03	1.786E+03
	Mean	1.761E+03	1.859E+03	1.857E+03	1.899E+03	1.979E+03	1.861E+03	1.986E+03	1.799E+03	2.013E+03	1.896E+03
	Std	2.673E+01	1.220E+02	1.382E+02	1.078E+02	1.433E+02	4.504E+01	1.606E+02	4.769E+01	4.962E+01	6.563E+01
	Rank	1	4	3	7	8	5	9	2	10	6
F17	Best	1.829E+03	2.548E+04	2.127E+04	3.547E+04	1.403E+04	4.258E+03	2.946E+04	9.219E+03	2.609E+05	3.787E+04
	Mean	1.832E+03	2.138E+05	1.588E+05	3.569E+05	8.507E+04	2.440E+04	1.360E+05	3.035E+04	8.193E+05	9.375E+04
	Std	1.782E+00	1.622E+05	1.296E+05	3.048E+05	6.734E+04	9.438E+03	1.196E+05	1.144E+04	3.118E+05	3.534E+04
	Rank	1	8	7	9	4	2	6	3	10	5
F18	Best	1.915E+03	1.941E+03	1.926E+03	2.435E+03	1.957E+03	2.083E+03	1.952E+03	1.927E+03	1.047E+04	4.135E+03
	Mean	1.925E+03	1.157E+04	8.475E+03	1.885E+04	2.016E+03	5.342E+03	1.138E+04	2.599E+03	2.905E+04	3.300E+04
	Std	2.765E+00	1.259E+04	1.125E+04	1.690E+04	5.646E+01	2.299E+03	1.117E+04	4.617E+02	1.090E+04	3.845E+04
	Rank	1	7	5	8	2	4	6	3	9	10
F19	Best	2.002E+03	2.030E+03	2.023E+03	2.047E+03	2.059E+03	2.105E+03	2.044E+03	2.032E+03	2.242E+03	2.148E+03
	Mean	2.048E+03	2.158E+03	2.138E+03	2.237E+03	2.336E+03	2.227E+03	2.329E+03	2.151E+03	2.472E+03	2.336E+03
	Std	4.935E+01	7.750E+01	7.715E+01	1.199E+02	1.416E+02	5.759E+01	1.558E+02	7.211E+01	7.264E+01	5.610E+01
	Rank	1	4	2	6	9	5	7	3	10	8
F20	Best	2.311E+03	2.318E+03	2.320E+03	2.336E+03	2.387E+03	2.224E+03	2.343E+03	2.317E+03	2.437E+03	2.354E+03
	Mean	2.335E+03	2.338E+03	2.343E+03	2.367E+03	2.443E+03	2.398E+03	2.392E+03	2.336E+03	2.465E+03	2.398E+03
	Std	2.717E+01	1.643E+01	1.344E+01	1.736E+01	2.208E+01	5.712E+01	2.348E+01	1.122E+01	1.067E+01	1.577E+01
	Rank	1	3	4	5	9	7	6	2	10	8
F21	Best	2.300E+03	2.300E+03	2.300E+03	2.303E+03	2.300E+03	2.353E+03	2.300E+03	2.300E+03	2.300E+03	2.332E+03
	Mean	2.300E+03	2.300E+03	2.528E+03	2.368E+03	2.301E+03	2.459E+03	2.337E+03	2.300E+03	2.300E+03	2.356E+03
	Std	2.002E-12	1.396E-01	8.062E+02	4.304E+02	1.449E+00	5.098E+01	2.608E+02	3.501E-01	2.495E-02	1.628E+01
	Rank	1	4	10	8	5	9	6	2	3	7
F22	Best	2.644E+03	2.665E+03	2.664E+03	2.685E+03	2.686E+03	2.711E+03	2.673E+03	2.658E+03	2.788E+03	2.724E+03
	Mean	2.664E+03	2.688E+03	2.694E+03	2.716E+03	2.824E+03	2.781E+03	2.759E+03	2.685E+03	2.817E+03	2.759E+03
	Std	9.273E+00	1.239E+01	1.342E+01	1.708E+01	4.613E+01	2.279E+01	3.562E+01	1.344E+01	9.514E+00	1.428E+01
	Rank	1	3	4	5	10	8	6	2	9	7
F23	Best	2.700E+03	2.831E+03	2.841E+03	2.854E+03	2.891E+03	2.909E+03	2.874E+03	2.833E+03	2.956E+03	2.884E+03
	Mean	2.805E+03	2.861E+03	2.872E+03	2.892E+03	2.988E+03	2.970E+03	2.939E+03	2.850E+03	2.989E+03	2.914E+03
	Std	4.683E+01	1.865E+01	1.795E+01	1.677E+01	4.976E+01	2.587E+01	3.521E+01	1.081E+01	1.110E+01	1.550E+01
	Rank	1	3	4	5	9	8	7	2	10	6
F24	Best	2.887E+03	2.883E+03	2.883E+03	2.884E+03	2.884E+03	2.965E+03	2.884E+03	2.883E+03	2.887E+03	2.919E+03
	Mean	2.887E+03	2.887E+03	2.887E+03	2.893E+03	2.908E+03	3.040E+03	2.892E+03	2.887E+03	2.887E+03	2.960E+03
	Std	5.577E-02	1.619E+00	5.646E+00	1.300E+01	2.303E+01	4.634E+01	1.408E+01	2.226E+00	4.089E-02	1.991E+01
	Rank	2	5	1	7	8	10	6	3	4	9
F25	Best	2.900E+03	2.801E+03	2.900E+03	2.809E+03	2.800E+03	3.077E+03	2.800E+03	2.803E+03	4.842E+03	3.091E+03
	Mean	3.707E+03	3.878E+03	4.027E+03	4.235E+03	3.250E+03	3.419E+03	4.542E+03	3.709E+03	5.125E+03	4.041E+03
	Std	1.809E+02	3.001E+02	2.693E+02	4.557E+02	7.368E+02	2.080E+02	1.018E+03	3.736E+02	9.730E+01	6.623E+02
	Rank	3	5	6	8	1	2	9	4	10	7
F26	Best	3.162E+03	3.184E+03	3.192E+03	3.201E+03	3.234E+03	3.203E+03	3.207E+03	3.186E+03	3.228E+03	3.211E+03
	Mean	3.196E+03	3.205E+03	3.210E+03	3.214E+03	3.272E+03	3.229E+03	3.247E+03	3.205E+03	3.237E+03	3.242E+03
	Std	1.829E+01	9.712E+00	6.839E+00	6.567E+00	2.650E+01	1.297E+01	2.331E+01	9.083E+00	3.466E+00	9.930E+00
	Rank	1	3	4	5	10	6	9	2	7	8
F27	Best	3.100E+03	3.194E+03	3.105E+03	3.208E+03	3.103E+03	3.271E+03	3.181E+03	3.111E+03	3.193E+03	3.238E+03
	Mean	3.128E+03	3.224E+03	3.210E+03	3.249E+03	3.222E+03	3.405E+03	3.213E+03	3.196E+03	3.206E+03	3.305E+03
	Std	4.814E+01	1.856E+01	2.390E+01	2.639E+01	3.401E+01	6.941E+01	1.792E+01	2.822E+01	8.642E+00	3.204E+01
	Rank	1	7	4	8	6	10	5	2	3	9
F28	Best	3.259E+03	3.287E+03	3.320E+03	3.360E+03	3.558E+03	3.520E+03	3.372E+03	3.356E+03	3.703E+03	3.627E+03
	Mean	3.409E+03	3.459E+03	3.438E+03	3.595E+03	3.949E+03	3.693E+03	3.713E+03	3.470E+03	3.877E+03	3.820E+03
	Std	7.564E+01	1.169E+02	1.005E+02	1.328E+02	1.786E+02	8.514E+01	1.926E+02	7.220E+01	7.418E+01	1.032E+02
	Rank	1	3	2	5	10	6	7	4	9	8
F29	Best	5.039E+03	6.735E+03	5.110E+03	5.880E+04	9.445E+03	2.187E+04	6.857E+03	7.007E+03	8.980E+04	6.076E+04
	Mean	5.252E+03	1.173E+04	8.506E+03	3.055E+05	1.539E+04	7.169E+04	1.215E+04	1.745E+04	2.166E+05	2.512E+05
	Std	1.572E+02	5.065E+03	4.083E+03	2.539E+05	4.647E+03	4.281E+04	3.852E+03	1.050E+04	5.162E+04	2.143E+05
	Rank	1	3	2	10	5	7	4	6	8	9

**Table A3:** Comparison results on of MSAO-EDA and its competitors on CEC2017 (50 D).

No.	Metric	MSAO-EDA	SAO	EO	RIME	AFDBARO	CSOAOA	MRFO	EOSMA	JADE	CFOA
F1	Best	1.000E+02	2.478E+03	1.070E+02	1.198E+05	1.006E+02	7.267E+08	1.008E+02	1.003E+02	1.075E+04	3.222E+07
	Mean	1.779E+02	1.001E+04	3.438E+03	4.932E+05	1.048E+02	2.264E+09	3.153E+03	4.016E+03	2.419E+04	1.069E+08
	Std	5.345E+02	5.401E+03	4.068E+03	2.948E+05	6.561E+00	9.431E+08	3.807E+03	4.799E+03	9.062E+03	5.444E+07
	Rank	2	6	4	8	1	10	3	5	7	9
F2	Best	3.000E+02	7.930E+03	3.618E+02	4.345E+02	1.385E+04	1.614E+04	5.479E+02	1.126E+03	3.834E+04	6.350E+03
	Mean	3.000E+02	1.579E+04	5.329E+02	7.048E+02	3.839E+04	2.617E+04	1.403E+03	2.186E+03	5.624E+04	1.675E+04
	Std	9.423E-12	5.072E+03	1.692E+02	1.595E+02	1.184E+04	4.726E+03	5.489E+02	6.106E+02	7.072E+03	4.882E+03
	Rank	1	6	2	3	9	8	4	5	10	7
F3	Best	4.000E+02	4.721E+02	4.679E+02	4.207E+02	4.512E+02	5.852E+02	4.001E+02	4.451E+02	4.870E+02	4.950E+02
	Mean	4.458E+02	4.958E+02	4.938E+02	4.970E+02	4.888E+02	7.730E+02	4.896E+02	4.916E+02	5.063E+02	5.380E+02
	Std	3.143E+01	1.235E+01	1.604E+01	2.057E+01	1.781E+01	1.811E+02	2.860E+01	1.814E+01	1.242E+01	1.831E+01
	Rank	1	6	5	7	2	10	3	4	8	9
F4	Best	5.099E+02	5.187E+02	5.219E+02	5.311E+02	5.802E+02	5.954E+02	5.448E+02	5.159E+02	6.388E+02	5.953E+02
	Mean	5.370E+02	5.380E+02	5.470E+02	5.595E+02	6.347E+02	6.315E+02	6.162E+02	5.354E+02	6.693E+02	6.243E+02
	Std	2.742E+01	1.143E+01	1.399E+01	1.784E+01	2.185E+01	1.616E+01	3.647E+01	1.038E+01	8.972E+00	1.531E+01
	Rank	2	3	4	5	9	8	6	1	10	7
F5	Best	6.000E+02	6.000E+02	6.000E+02	6.003E+02	6.000E+02	6.060E+02	6.000E+02	6.000E+02	6.001E+02	6.098E+02
	Mean	6.000E+02	6.001E+02	6.000E+02	6.010E+02	6.001E+02	6.097E+02	6.032E+02	6.000E+02	6.002E+02	6.152E+02
	Std	7.570E-08	3.418E-02	1.234E-03	5.131E-01	1.736E-01	2.012E+00	5.695E+00	1.400E-02	1.284E-02	2.608E+00
	Rank	1	4	2	7	5	9	8	3	6	10
F6	Best	7.439E+02	7.536E+02	7.526E+02	7.674E+02	8.217E+02	8.814E+02	7.842E+02	7.485E+02	8.635E+02	8.228E+02
	Mean	8.054E+02	8.401E+02	7.714E+02	8.012E+02	8.823E+02	9.353E+02	8.787E+02	7.664E+02	9.058E+02	8.763E+02
	Std	5.637E+01	4.815E+01	1.250E+01	1.613E+01	3.144E+01	2.220E+01	5.356E+01	1.121E+01	1.028E+01	2.327E+01
	Rank	4	5	2	3	8	10	7	1	9	6
F7	Best	8.109E+02	8.170E+02	8.179E+02	8.274E+02	8.773E+02	8.801E+02	8.587E+02	8.229E+02	9.370E+02	8.637E+02
	Mean	8.343E+02	8.352E+02	8.479E+02	8.586E+02	9.315E+02	9.056E+02	9.187E+02	8.370E+02	9.697E+02	9.033E+02
	Std	2.612E+01	1.039E+01	1.450E+01	1.335E+01	2.221E+01	1.101E+01	2.716E+01	9.532E+00	8.866E+00	1.605E+01
	Rank	1	2	4	5	9	7	8	3	10	6
F8	Best	9.000E+02	9.000E+02	9.000E+02	9.028E+02	9.296E+02	1.005E+03	9.191E+02	9.001E+02	9.001E+02	1.113E+03
	Mean	9.000E+02	9.007E+02	9.009E+02	9.593E+02	1.513E+03	1.492E+03	2.099E+03	9.017E+02	9.002E+02	1.315E+03
	Std	0.000E+00	1.677E+00	9.578E-01	6.762E+01	5.524E+02	5.079E+02	1.091E+03	1.757E+00	4.592E-02	1.517E+02
	Rank	1	3	4	6	9	8	10	5	2	7
F9	Best	2.345E+03	1.765E+03	1.908E+03	2.445E+03	3.329E+03	4.523E+03	2.909E+03	3.168E+03	6.964E+03	3.977E+03
	Mean	6.151E+03	3.297E+03	4.262E+03	3.890E+03	4.166E+03	5.179E+03	4.367E+03	4.422E+03	7.925E+03	5.447E+03
	Std	1.879E+03	1.326E+03	7.983E+02	5.648E+02	4.076E+02	2.418E+02	5.786E+02	6.354E+02	3.275E+02	6.523E+02
	Rank	9	1	4	2	3	7	5	6	10	8
F10	Best	1.103E+03	1.111E+03	1.111E+03	1.157E+03	1.158E+03	1.204E+03	1.144E+03	1.111E+03	1.200E+03	1.277E+03
	Mean	1.121E+03	1.140E+03	1.154E+03	1.251E+03	1.237E+03	1.303E+03	1.202E+03	1.153E+03	1.237E+03	1.389E+03
	Std	2.375E+01	2.644E+01	3.103E+01	4.486E+01	3.439E+01	5.609E+01	3.839E+01	2.981E+01	1.648E+01	7.075E+01
	Rank	1	2	4	8	7	9	5	3	6	10
F11	Best	1.201E+03	1.053E+05	2.720E+04	3.390E+05	6.402E+04	3.868E+06	2.798E+04	1.503E+04	9.189E+05	4.932E+05
	Mean	1.450E+03	8.211E+05	1.074E+05	6.322E+06	8.256E+05	2.214E+07	2.714E+05	6.292E+04	2.111E+06	4.811E+06
	Std	1.866E+02	5.887E+05	1.145E+05	4.809E+06	5.323E+05	1.177E+07	2.345E+05	3.705E+04	4.913E+05	2.938E+06
	Rank	1	5	3	9	6	10	4	2	7	8
F12	Best	1.338E+03	1.578E+03	2.245E+03	1.393E+04	2.016E+03	9.479E+03	1.377E+03	1.424E+03	6.507E+04	1.493E+04
	Mean	1.365E+03	2.670E+04	2.466E+04	8.147E+04	7.456E+03	1.758E+05	1.664E+04	1.141E+04	1.633E+05	2.627E+04
	Std	1.828E+01	2.210E+04	2.003E+04	5.965E+04	1.141E+04	2.094E+05	1.737E+04	1.003E+04	4.352E+04	1.187E+04
	Rank	1	7	5	8	2	10	4	3	9	6
F13	Best	1.427E+03	2.078E+03	2.334E+03	2.806E+03	1.508E+03	1.469E+03	1.761E+03	1.537E+03	3.705E+03	1.922E+03
	Mean	1.452E+03	1.717E+04	7.685E+03	1.262E+04	2.034E+03	1.505E+03	4.495E+03	1.596E+03	9.446E+03	7.290E+03
	Std	1.001E+01	1.203E+04	5.448E+03	8.507E+03	9.574E+02	2.241E+01	3.881E+03	3.878E+01	3.143E+03	4.984E+03
	Rank	1	10	7	9	4	2	5	3	8	6
F14	Best	1.520E+03	1.563E+03	1.530E+03	4.083E+03	1.594E+03	1.922E+03	1.596E+03	1.678E+03	1.100E+04	2.467E+03
	Mean	1.530E+03	4.061E+03	5.361E+03	1.879E+04	2.373E+03	2.398E+03	9.906E+03	2.261E+03	3.148E+04	1.412E+04
	Std	5.067E+00	3.242E+03	4.659E+03	1.486E+04	3.898E+03	3.310E+02	8.492E+03	5.149E+02	9.931E+03	9.941E+03
	Rank	1	5	6	9	3	4	7	2	10	8
F15	Best	1.616E+03	1.618E+03	1.619E+03	1.663E+03	2.029E+03	2.161E+03	1.977E+03	1.621E+03	2.474E+03	2.059E+03
	Mean	2.062E+03	2.068E+03	2.171E+03	2.253E+03	2.541E+03	2.384E+03	2.468E+03	1.982E+03	2.924E+03	2.514E+03
	Std	3.397E+02	2.326E+02	2.758E+02	2.576E+02	2.474E+02	1.190E+02	2.318E+02	1.865E+02	1.538E+02	2.110E+02
	Rank	2	3	4	5	9	6	7	1	10	8
F16	Best	1.731E+03	1.738E+03	1.729E+03	1.757E+03	1.766E+03	1.778E+03	1.760E+03	1.739E+03	1.910E+03	1.786E+03
	Mean	1.761E+03	1.859E+03	1.857E+03	1.899E+03	1.979E+03	1.861E+03	1.986E+03	1.799E+03	2.013E+03	1.896E+03
	Std	2.673E+01	1.220E+02	1.382E+02	1.078E+02	1.433E+02	4.504E+01	1.606E+02	4.769E+01	4.962E+01	6.563E+01
	Rank	1	4	3	7	8	5	9	2	10	6
F17	Best	1.829E+03	2.548E+04	2.127E+04	3.547E+04	1.403E+04	4.258E+03	2.946E+04	9.219E+03	2.609E+05	3.787E+04
	Mean	1.832E+03	2.138E+05	1.588E+05	3.569E+05	8.507E+04	2.440E+04	1.360E+05	3.035E+04	8.193E+05	9.375E+04
	Std	1.782E+00	1.622E+05	1.296E+05	3.048E+05	6.734E+04	9.438E+03	1.196E+05	1.144E+04	3.118E+05	3.534E+04
	Rank	1	8	7	9	4	2	6	3	10	5
F18	Best	1.915E+03	1.941E+03	1.926E+03	2.435E+03	1.957E+03	2.083E+03	1.952E+03	1.927E+03	1.047E+04	4.135E+03
	Mean	1.925E+03	1.157E+04	8.475E+03	1.885E+04	2.016E+03	5.342E+03	1.138E+04	2.599E+03	2.905E+04	3.300E+04
	Std	2.765E+00	1.259E+04	1.125E+04	1.690E+04	5.646E+01	2.299E+03	1.117E+04	4.617E+02	1.090E+04	3.845E+04
	Rank	1	7	5	8	2	4	6	3	9	10
F19	Best	2.002E+03	2.030E+03	2.023E+03	2.047E+03	2.059E+03	2.105E+03	2.044E+03	2.032E+03	2.242E+03	2.148E+03
	Mean	2.048E+03	2.158E+03	2.138E+03	2.237E+03	2.336E+03	2.227E+03	2.329E+03	2.151E+03	2.472E+03	2.336E+03
	Std	4.935E+01	7.750E+01	7.715E+01	1.199E+02	1.416E+02	5.759E+01	1.558E+02	7.211E+01	7.264E+01	5.610E+01
	Rank	1	4	2	6	9	5	7	3	10	8
F20	Best	2.311E+03	2.318E+03	2.320E+03	2.336E+03	2.387E+03	2.224E+03	2.343E+03	2.317E+03	2.437E+03	2.354E+03
	Mean	2.335E+03	2.338E+03	2.343E+03	2.367E+03	2.443E+03	2.398E+03	2.392E+03	2.336E+03	2.465E+03	2.398E+03
	Std	2.717E+01	1.643E+01	1.344E+01	1.736E+01	2.208E+01	5.712E+01	2.348E+01	1.122E+01	1.067E+01	1.577E+01
	Rank	1	3	4	5	9	7	6	2	10	8
F21	Best	2.300E+03	2.300E+03	2.300E+03	2.303E+03	2.300E+03	2.353E+03	2.300E+03	2.300E+03	2.300E+03	2.332E+03
	Mean	2.300E+03	2.300E+03	2.528E+03	2.368E+03	2.301E+03	2.459E+03	2.337E+03	2.300E+03	2.300E+03	2.356E+03
	Std	2.002E-12	1.396E-01	8.062E+02	4.304E+02	1.449E+00	5.098E+01	2.608E+02	3.501E-01	2.495E-02	1.628E+01
	Rank	1	4	10	8	5	9	6	2	3	7
F22	Best	2.644E+03	2.665E+03	2.664E+03	2.685E+03	2.686E+03	2.711E+03	2.673E+03	2.658E+03	2.788E+03	2.724E+03
	Mean	2.664E+03	2.688E+03	2.694E+03	2.716E+03	2.824E+03	2.781E+03	2.759E+03	2.685E+03	2.817E+03	2.759E+03
	Std	9.273E+00	1.239E+01	1.342E+01	1.708E+01	4.613E+01	2.279E+01	3.562E+01	1.344E+01	9.514E+00	1.428E+01
	Rank	1	3	4	5	10	8	6	2	9	7
F23	Best	2.700E+03	2.831E+03	2.841E+03	2.854E+03	2.891E+03	2.909E+03	2.874E+03	2.833E+03	2.956E+03	2.884E+03
	Mean	2.805E+03	2.861E+03	2.872E+03	2.892E+03	2.988E+03	2.970E+03	2.939E+03	2.850E+03	2.989E+03	2.914E+03
	Std	4.683E+01	1.865E+01	1.795E+01	1.677E+01	4.976E+01	2.587E+01	3.521E+01	1.081E+01	1.110E+01	1.550E+01
	Rank	1	3	4	5	9	8	7	2	10	6
F24	Best	2.887E+03	2.883E+03	2.883E+03	2.884E+03	2.884E+03	2.965E+03	2.884E+03	2.883E+03	2.887E+03	2.919E+03
	Mean	2.887E+03	2.887E+03	2.887E+03	2.893E+03	2.908E+03	3.040E+03	2.892E+03	2.887E+03	2.887E+03	2.960E+03
	Std	5.577E-02	1.619E+00	5.646E+00	1.300E+01	2.303E+01	4.634E+01	1.408E+01	2.226E+00	4.089E-02	1.991E+01
	Rank	2	5	1	7	8	10	6	3	4	9
F25	Best	2.900E+03	2.801E+03	2.900E+03	2.809E+03	2.800E+03	3.077E+03	2.800E+03	2.803E+03	4.842E+03	3.091E+03
	Mean	3.707E+03	3.878E+03	4.027E+03	4.235E+03	3.250E+03	3.419E+03	4.542E+03	3.709E+03	5.125E+03	4.041E+03
	Std	1.809E+02	3.001E+02	2.693E+02	4.557E+02	7.368E+02	2.080E+02	1.018E+03	3.736E+02	9.730E+01	6.623E+02
	Rank	3	5	6	8	1	2	9	4	10	7
F26	Best	3.162E+03	3.184E+03	3.192E+03	3.201E+03	3.234E+03	3.203E+03	3.207E+03	3.186E+03	3.228E+03	3.211E+03
	Mean	3.196E+03	3.205E+03	3.210E+03	3.214E+03	3.272E+03	3.229E+03	3.247E+03	3.205E+03	3.237E+03	3.242E+03
	Std	1.829E+01	9.712E+00	6.839E+00	6.567E+00	2.650E+01	1.297E+01	2.331E+01	9.083E+00	3.466E+00	9.930E+00
	Rank	1	3	4	5	10	6	9	2	7	8
F27	Best	3.100E+03	3.194E+03	3.105E+03	3.208E+03	3.103E+03	3.271E+03	3.181E+03	3.111E+03	3.193E+03	3.238E+03
	Mean	3.128E+03	3.224E+03	3.210E+03	3.249E+03	3.222E+03	3.405E+03	3.213E+03	3.196E+03	3.206E+03	3.305E+03
	Std	4.814E+01	1.856E+01	2.390E+01	2.639E+01	3.401E+01	6.941E+01	1.792E+01	2.822E+01	8.642E+00	3.204E+01
	Rank	1	7	4	8	6	10	5	2	3	9
F28	Best	3.259E+03	3.287E+03	3.320E+03	3.360E+03	3.558E+03	3.520E+03	3.372E+03	3.356E+03	3.703E+03	3.627E+03
	Mean	3.409E+03	3.459E+03	3.438E+03	3.595E+03	3.949E+03	3.693E+03	3.713E+03	3.470E+03	3.877E+03	3.820E+03
	Std	7.564E+01	1.169E+02	1.005E+02	1.328E+02	1.786E+02	8.514E+01	1.926E+02	7.220E+01	7.418E+01	1.032E+02
	Rank	1	3	2	5	10	6	7	4	9	8
F29	Best	5.039E+03	6.735E+03	5.110E+03	5.880E+04	9.445E+03	2.187E+04	6.857E+03	7.007E+03	8.980E+04	6.076E+04
	Mean	5.252E+03	1.173E+04	8.506E+03	3.055E+05	1.539E+04	7.169E+04	1.215E+04	1.745E+04	2.166E+05	2.512E+05
	Std	1.572E+02	5.065E+03	4.083E+03	2.539E+05	4.647E+03	4.281E+04	3.852E+03	1.050E+04	5.162E+04	2.143E+05
	Rank	1	3	2	10	5	7	4	6	8	9

**Table A4:** Comparison results on of MSAO-EDA and its competitors on CEC2017 (100 D).

No.	Metric	MSAO-EDA	SAO	EO	RIME	AFDBARO	CSOAOA	MRFO	EOSMA	JADE	CFOA
F1	Best	1.000E+02	2.478E+03	1.070E+02	1.198E+05	1.006E+02	7.267E+08	1.008E+02	1.003E+02	1.075E+04	3.222E+07
	Mean	1.779E+02	1.001E+04	3.438E+03	4.932E+05	1.048E+02	2.264E+09	3.153E+03	4.016E+03	2.419E+04	1.069E+08
	Std	5.345E+02	5.401E+03	4.068E+03	2.948E+05	6.561E+00	9.431E+08	3.807E+03	4.799E+03	9.062E+03	5.444E+07
	Rank	2	6	4	8	1	10	3	5	7	9
F2	Best	3.000E+02	7.930E+03	3.618E+02	4.345E+02	1.385E+04	1.614E+04	5.479E+02	1.126E+03	3.834E+04	6.350E+03
	Mean	3.000E+02	1.579E+04	5.329E+02	7.048E+02	3.839E+04	2.617E+04	1.403E+03	2.186E+03	5.624E+04	1.675E+04
	Std	9.423E-12	5.072E+03	1.692E+02	1.595E+02	1.184E+04	4.726E+03	5.489E+02	6.106E+02	7.072E+03	4.882E+03
	Rank	1	6	2	3	9	8	4	5	10	7
F3	Best	4.000E+02	4.721E+02	4.679E+02	4.207E+02	4.512E+02	5.852E+02	4.001E+02	4.451E+02	4.870E+02	4.950E+02
	Mean	4.458E+02	4.958E+02	4.938E+02	4.970E+02	4.888E+02	7.730E+02	4.896E+02	4.916E+02	5.063E+02	5.380E+02
	Std	3.143E+01	1.235E+01	1.604E+01	2.057E+01	1.781E+01	1.811E+02	2.860E+01	1.814E+01	1.242E+01	1.831E+01
	Rank	1	6	5	7	2	10	3	4	8	9
F4	Best	5.099E+02	5.187E+02	5.219E+02	5.311E+02	5.802E+02	5.954E+02	5.448E+02	5.159E+02	6.388E+02	5.953E+02
	Mean	5.370E+02	5.380E+02	5.470E+02	5.595E+02	6.347E+02	6.315E+02	6.162E+02	5.354E+02	6.693E+02	6.243E+02
	Std	2.742E+01	1.143E+01	1.399E+01	1.784E+01	2.185E+01	1.616E+01	3.647E+01	1.038E+01	8.972E+00	1.531E+01
	Rank	2	3	4	5	9	8	6	1	10	7
F5	Best	6.000E+02	6.000E+02	6.000E+02	6.003E+02	6.000E+02	6.060E+02	6.000E+02	6.000E+02	6.001E+02	6.098E+02
	Mean	6.000E+02	6.001E+02	6.000E+02	6.010E+02	6.001E+02	6.097E+02	6.032E+02	6.000E+02	6.002E+02	6.152E+02
	Std	7.570E-08	3.418E-02	1.234E-03	5.131E-01	1.736E-01	2.012E+00	5.695E+00	1.400E-02	1.284E-02	2.608E+00
	Rank	1	4	2	7	5	9	8	3	6	10
F6	Best	7.439E+02	7.536E+02	7.526E+02	7.674E+02	8.217E+02	8.814E+02	7.842E+02	7.485E+02	8.635E+02	8.228E+02
	Mean	8.054E+02	8.401E+02	7.714E+02	8.012E+02	8.823E+02	9.353E+02	8.787E+02	7.664E+02	9.058E+02	8.763E+02
	Std	5.637E+01	4.815E+01	1.250E+01	1.613E+01	3.144E+01	2.220E+01	5.356E+01	1.121E+01	1.028E+01	2.327E+01
	Rank	4	5	2	3	8	10	7	1	9	6
F7	Best	8.109E+02	8.170E+02	8.179E+02	8.274E+02	8.773E+02	8.801E+02	8.587E+02	8.229E+02	9.370E+02	8.637E+02
	Mean	8.343E+02	8.352E+02	8.479E+02	8.586E+02	9.315E+02	9.056E+02	9.187E+02	8.370E+02	9.697E+02	9.033E+02
	Std	2.612E+01	1.039E+01	1.450E+01	1.335E+01	2.221E+01	1.101E+01	2.716E+01	9.532E+00	8.866E+00	1.605E+01
	Rank	1	2	4	5	9	7	8	3	10	6
F8	Best	9.000E+02	9.000E+02	9.000E+02	9.028E+02	9.296E+02	1.005E+03	9.191E+02	9.001E+02	9.001E+02	1.113E+03
	Mean	9.000E+02	9.007E+02	9.009E+02	9.593E+02	1.513E+03	1.492E+03	2.099E+03	9.017E+02	9.002E+02	1.315E+03
	Std	0.000E+00	1.677E+00	9.578E-01	6.762E+01	5.524E+02	5.079E+02	1.091E+03	1.757E+00	4.592E-02	1.517E+02
	Rank	1	3	4	6	9	8	10	5	2	7
F9	Best	2.345E+03	1.765E+03	1.908E+03	2.445E+03	3.329E+03	4.523E+03	2.909E+03	3.168E+03	6.964E+03	3.977E+03
	Mean	6.151E+03	3.297E+03	4.262E+03	3.890E+03	4.166E+03	5.179E+03	4.367E+03	4.422E+03	7.925E+03	5.447E+03
	Std	1.879E+03	1.326E+03	7.983E+02	5.648E+02	4.076E+02	2.418E+02	5.786E+02	6.354E+02	3.275E+02	6.523E+02
	Rank	9	1	4	2	3	7	5	6	10	8
F10	Best	1.103E+03	1.111E+03	1.111E+03	1.157E+03	1.158E+03	1.204E+03	1.144E+03	1.111E+03	1.200E+03	1.277E+03
	Mean	1.121E+03	1.140E+03	1.154E+03	1.251E+03	1.237E+03	1.303E+03	1.202E+03	1.153E+03	1.237E+03	1.389E+03
	Std	2.375E+01	2.644E+01	3.103E+01	4.486E+01	3.439E+01	5.609E+01	3.839E+01	2.981E+01	1.648E+01	7.075E+01
	Rank	1	2	4	8	7	9	5	3	6	10
F11	Best	1.201E+03	1.053E+05	2.720E+04	3.390E+05	6.402E+04	3.868E+06	2.798E+04	1.503E+04	9.189E+05	4.932E+05
	Mean	1.450E+03	8.211E+05	1.074E+05	6.322E+06	8.256E+05	2.214E+07	2.714E+05	6.292E+04	2.111E+06	4.811E+06
	Std	1.866E+02	5.887E+05	1.145E+05	4.809E+06	5.323E+05	1.177E+07	2.345E+05	3.705E+04	4.913E+05	2.938E+06
	Rank	1	5	3	9	6	10	4	2	7	8
F12	Best	1.338E+03	1.578E+03	2.245E+03	1.393E+04	2.016E+03	9.479E+03	1.377E+03	1.424E+03	6.507E+04	1.493E+04
	Mean	1.365E+03	2.670E+04	2.466E+04	8.147E+04	7.456E+03	1.758E+05	1.664E+04	1.141E+04	1.633E+05	2.627E+04
	Std	1.828E+01	2.210E+04	2.003E+04	5.965E+04	1.141E+04	2.094E+05	1.737E+04	1.003E+04	4.352E+04	1.187E+04
	Rank	1	7	5	8	2	10	4	3	9	6
F13	Best	1.427E+03	2.078E+03	2.334E+03	2.806E+03	1.508E+03	1.469E+03	1.761E+03	1.537E+03	3.705E+03	1.922E+03
	Mean	1.452E+03	1.717E+04	7.685E+03	1.262E+04	2.034E+03	1.505E+03	4.495E+03	1.596E+03	9.446E+03	7.290E+03
	Std	1.001E+01	1.203E+04	5.448E+03	8.507E+03	9.574E+02	2.241E+01	3.881E+03	3.878E+01	3.143E+03	4.984E+03
	Rank	1	10	7	9	4	2	5	3	8	6
F14	Best	1.520E+03	1.563E+03	1.530E+03	4.083E+03	1.594E+03	1.922E+03	1.596E+03	1.678E+03	1.100E+04	2.467E+03
	Mean	1.530E+03	4.061E+03	5.361E+03	1.879E+04	2.373E+03	2.398E+03	9.906E+03	2.261E+03	3.148E+04	1.412E+04
	Std	5.067E+00	3.242E+03	4.659E+03	1.486E+04	3.898E+03	3.310E+02	8.492E+03	5.149E+02	9.931E+03	9.941E+03
	Rank	1	5	6	9	3	4	7	2	10	8
F15	Best	1.616E+03	1.618E+03	1.619E+03	1.663E+03	2.029E+03	2.161E+03	1.977E+03	1.621E+03	2.474E+03	2.059E+03
	Mean	2.062E+03	2.068E+03	2.171E+03	2.253E+03	2.541E+03	2.384E+03	2.468E+03	1.982E+03	2.924E+03	2.514E+03
	Std	3.397E+02	2.326E+02	2.758E+02	2.576E+02	2.474E+02	1.190E+02	2.318E+02	1.865E+02	1.538E+02	2.110E+02
	Rank	2	3	4	5	9	6	7	1	10	8
F16	Best	1.731E+03	1.738E+03	1.729E+03	1.757E+03	1.766E+03	1.778E+03	1.760E+03	1.739E+03	1.910E+03	1.786E+03
	Mean	1.761E+03	1.859E+03	1.857E+03	1.899E+03	1.979E+03	1.861E+03	1.986E+03	1.799E+03	2.013E+03	1.896E+03
	Std	2.673E+01	1.220E+02	1.382E+02	1.078E+02	1.433E+02	4.504E+01	1.606E+02	4.769E+01	4.962E+01	6.563E+01
	Rank	1	4	3	7	8	5	9	2	10	6
F17	Best	1.829E+03	2.548E+04	2.127E+04	3.547E+04	1.403E+04	4.258E+03	2.946E+04	9.219E+03	2.609E+05	3.787E+04
	Mean	1.832E+03	2.138E+05	1.588E+05	3.569E+05	8.507E+04	2.440E+04	1.360E+05	3.035E+04	8.193E+05	9.375E+04
	Std	1.782E+00	1.622E+05	1.296E+05	3.048E+05	6.734E+04	9.438E+03	1.196E+05	1.144E+04	3.118E+05	3.534E+04
	Rank	1	8	7	9	4	2	6	3	10	5
F18	Best	1.915E+03	1.941E+03	1.926E+03	2.435E+03	1.957E+03	2.083E+03	1.952E+03	1.927E+03	1.047E+04	4.135E+03
	Mean	1.925E+03	1.157E+04	8.475E+03	1.885E+04	2.016E+03	5.342E+03	1.138E+04	2.599E+03	2.905E+04	3.300E+04
	Std	2.765E+00	1.259E+04	1.125E+04	1.690E+04	5.646E+01	2.299E+03	1.117E+04	4.617E+02	1.090E+04	3.845E+04
	Rank	1	7	5	8	2	4	6	3	9	10
F19	Best	2.002E+03	2.030E+03	2.023E+03	2.047E+03	2.059E+03	2.105E+03	2.044E+03	2.032E+03	2.242E+03	2.148E+03
	Mean	2.048E+03	2.158E+03	2.138E+03	2.237E+03	2.336E+03	2.227E+03	2.329E+03	2.151E+03	2.472E+03	2.336E+03
	Std	4.935E+01	7.750E+01	7.715E+01	1.199E+02	1.416E+02	5.759E+01	1.558E+02	7.211E+01	7.264E+01	5.610E+01
	Rank	1	4	2	6	9	5	7	3	10	8
F20	Best	2.311E+03	2.318E+03	2.320E+03	2.336E+03	2.387E+03	2.224E+03	2.343E+03	2.317E+03	2.437E+03	2.354E+03
	Mean	2.335E+03	2.338E+03	2.343E+03	2.367E+03	2.443E+03	2.398E+03	2.392E+03	2.336E+03	2.465E+03	2.398E+03
	Std	2.717E+01	1.643E+01	1.344E+01	1.736E+01	2.208E+01	5.712E+01	2.348E+01	1.122E+01	1.067E+01	1.577E+01
	Rank	1	3	4	5	9	7	6	2	10	8
F21	Best	2.300E+03	2.300E+03	2.300E+03	2.303E+03	2.300E+03	2.353E+03	2.300E+03	2.300E+03	2.300E+03	2.332E+03
	Mean	2.300E+03	2.300E+03	2.528E+03	2.368E+03	2.301E+03	2.459E+03	2.337E+03	2.300E+03	2.300E+03	2.356E+03
	Std	2.002E-12	1.396E-01	8.062E+02	4.304E+02	1.449E+00	5.098E+01	2.608E+02	3.501E-01	2.495E-02	1.628E+01
	Rank	1	4	10	8	5	9	6	2	3	7
F22	Best	2.644E+03	2.665E+03	2.664E+03	2.685E+03	2.686E+03	2.711E+03	2.673E+03	2.658E+03	2.788E+03	2.724E+03
	Mean	2.664E+03	2.688E+03	2.694E+03	2.716E+03	2.824E+03	2.781E+03	2.759E+03	2.685E+03	2.817E+03	2.759E+03
	Std	9.273E+00	1.239E+01	1.342E+01	1.708E+01	4.613E+01	2.279E+01	3.562E+01	1.344E+01	9.514E+00	1.428E+01
	Rank	1	3	4	5	10	8	6	2	9	7
F23	Best	2.700E+03	2.831E+03	2.841E+03	2.854E+03	2.891E+03	2.909E+03	2.874E+03	2.833E+03	2.956E+03	2.884E+03
	Mean	2.805E+03	2.861E+03	2.872E+03	2.892E+03	2.988E+03	2.970E+03	2.939E+03	2.850E+03	2.989E+03	2.914E+03
	Std	4.683E+01	1.865E+01	1.795E+01	1.677E+01	4.976E+01	2.587E+01	3.521E+01	1.081E+01	1.110E+01	1.550E+01
	Rank	1	3	4	5	9	8	7	2	10	6
F24	Best	2.887E+03	2.883E+03	2.883E+03	2.884E+03	2.884E+03	2.965E+03	2.884E+03	2.883E+03	2.887E+03	2.919E+03
	Mean	2.887E+03	2.887E+03	2.887E+03	2.893E+03	2.908E+03	3.040E+03	2.892E+03	2.887E+03	2.887E+03	2.960E+03
	Std	5.577E-02	1.619E+00	5.646E+00	1.300E+01	2.303E+01	4.634E+01	1.408E+01	2.226E+00	4.089E-02	1.991E+01
	Rank	2	5	1	7	8	10	6	3	4	9
F25	Best	2.900E+03	2.801E+03	2.900E+03	2.809E+03	2.800E+03	3.077E+03	2.800E+03	2.803E+03	4.842E+03	3.091E+03
	Mean	3.707E+03	3.878E+03	4.027E+03	4.235E+03	3.250E+03	3.419E+03	4.542E+03	3.709E+03	5.125E+03	4.041E+03
	Std	1.809E+02	3.001E+02	2.693E+02	4.557E+02	7.368E+02	2.080E+02	1.018E+03	3.736E+02	9.730E+01	6.623E+02
	Rank	3	5	6	8	1	2	9	4	10	7
F26	Best	3.162E+03	3.184E+03	3.192E+03	3.201E+03	3.234E+03	3.203E+03	3.207E+03	3.186E+03	3.228E+03	3.211E+03
	Mean	3.196E+03	3.205E+03	3.210E+03	3.214E+03	3.272E+03	3.229E+03	3.247E+03	3.205E+03	3.237E+03	3.242E+03
	Std	1.829E+01	9.712E+00	6.839E+00	6.567E+00	2.650E+01	1.297E+01	2.331E+01	9.083E+00	3.466E+00	9.930E+00
	Rank	1	3	4	5	10	6	9	2	7	8
F27	Best	3.100E+03	3.194E+03	3.105E+03	3.208E+03	3.103E+03	3.271E+03	3.181E+03	3.111E+03	3.193E+03	3.238E+03
	Mean	3.128E+03	3.224E+03	3.210E+03	3.249E+03	3.222E+03	3.405E+03	3.213E+03	3.196E+03	3.206E+03	3.305E+03
	Std	4.814E+01	1.856E+01	2.390E+01	2.639E+01	3.401E+01	6.941E+01	1.792E+01	2.822E+01	8.642E+00	3.204E+01
	Rank	1	7	4	8	6	10	5	2	3	9
F28	Best	3.259E+03	3.287E+03	3.320E+03	3.360E+03	3.558E+03	3.520E+03	3.372E+03	3.356E+03	3.703E+03	3.627E+03
	Mean	3.409E+03	3.459E+03	3.438E+03	3.595E+03	3.949E+03	3.693E+03	3.713E+03	3.470E+03	3.877E+03	3.820E+03
	Std	7.564E+01	1.169E+02	1.005E+02	1.328E+02	1.786E+02	8.514E+01	1.926E+02	7.220E+01	7.418E+01	1.032E+02
	Rank	1	3	2	5	10	6	7	4	9	8
F29	Best	5.039E+03	6.735E+03	5.110E+03	5.880E+04	9.445E+03	2.187E+04	6.857E+03	7.007E+03	8.980E+04	6.076E+04
	Mean	5.252E+03	1.173E+04	8.506E+03	3.055E+05	1.539E+04	7.169E+04	1.215E+04	1.745E+04	2.166E+05	2.512E+05
	Std	1.572E+02	5.065E+03	4.083E+03	2.539E+05	4.647E+03	4.281E+04	3.852E+03	1.050E+04	5.162E+04	2.143E+05
	Rank	1	3	2	10	5	7	4	6	8	9

**Table A5:** Wilcoxon rank sum results of MSAO-EDA and its competitors on CEC2017 (10 D).

No.	SAO	EO	RIME	AFDBARO	CSOAOA	MRFO	EOSMA	JADE	CFOA
F1	1.39E-20	1.39E-20	1.39E-20	1.39E-20	1.39E-20	1.39E-20	1.39E-20	1.39E-20	1.39E-20
F2	1.39E-20	1.39E-20	1.39E-20	1.39E-20	1.39E-20	1.39E-20	1.39E-20	1.39E-20	1.39E-20
F3	5.03E-17	3.30E-18	1.08E-15	6.72E-03	6.66E-06	5.47E-01	1.28E-08	3.30E-18	4.00E-06
F4	1.45E-03	9.00E-06	1.37E-08	1.90E-16	8.76E-14	1.56E-11	2.36E-01	4.58E-18	8.65E-13
F5	1.39E-20	1.24E-20	1.39E-20	1.39E-20	1.39E-20	1.39E-20	1.39E-20	1.39E-20	1.39E-20
F6	3.86E-02	2.37E-02	4.27E-06	1.30E-12	2.84E-13	3.30E-13	1.85E-01	4.24E-16	6.66E-06
F7	1.14E-06	4.28E-03	2.29E-06	1.54E-13	5.42E-05	1.53E-14	3.84E-01	3.65E-18	3.51E-04
F8	1.82E-03	1.48E-11	1.39E-20	1.39E-20	1.39E-20	1.39E-20	2.20E-18	1.39E-20	1.39E-20
F9	8.83E-01	3.09E-01	1.16E-01	1.12E-02	6.77E-02	1.85E-02	1.25E-02	7.91E-17	2.08E-05
F10	1.49E-15	2.23E-10	1.34E-17	3.30E-18	3.72E-18	1.18E-16	2.45E-13	3.30E-18	3.30E-18
F11	3.30E-18	3.30E-18	3.30E-18	3.30E-18	3.30E-18	8.43E-18	3.30E-18	3.30E-18	3.30E-18
F12	3.30E-18	3.30E-18	3.30E-18	1.34E-17	9.26E-13	3.30E-18	1.69E-17	3.30E-18	3.30E-18
F13	7.36E-09	1.01E-17	3.75E-14	5.48E-03	1.54E-02	3.30E-18	3.36E-08	1.63E-14	4.18E-18
F14	3.30E-18	3.30E-18	3.30E-18	3.30E-18	4.18E-18	3.30E-18	3.30E-18	3.30E-18	3.30E-18
F15	1.10E-06	4.08E-10	2.43E-10	4.27E-06	3.09E-06	1.40E-06	1.07E-04	1.21E-10	4.08E-10
F16	8.87E-03	6.58E-04	6.28E-02	1.79E-01	3.30E-06	3.85E-05	1.04E-02	5.96E-13	1.72E-13
F17	3.30E-18	3.30E-18	3.30E-18	7.75E-16	4.44E-10	3.30E-18	3.30E-18	3.30E-18	3.30E-18
F18	3.30E-18	3.30E-18	3.30E-18	9.38E-17	4.14E-15	3.30E-18	3.30E-18	3.30E-18	3.30E-18
F19	4.93E-09	2.46E-06	2.47E-07	2.27E-03	6.68E-17	1.96E-05	2.23E-06	8.40E-13	1.20E-17
F20	3.95E-03	9.92E-06	4.30E-03	2.44E-02	4.21E-02	1.19E-02	2.12E-02	5.90E-03	2.44E-02
F21	3.94E-13	5.63E-13	3.83E-11	6.30E-06	8.32E-02	5.17E-10	1.16E-01	1.48E-19	3.78E-10
F22	2.75E-11	5.92E-11	2.47E-14	1.31E-16	1.72E-07	3.29E-13	1.02E-01	5.59E-18	8.04E-10
F23	2.77E-03	8.90E-08	5.50E-06	2.10E-12	1.90E-11	1.44E-05	2.96E-03	1.48E-01	5.91E-11
F24	1.80E-06	1.07E-07	5.03E-05	1.14E-01	3.95E-01	1.46E-03	1.72E-01	9.39E-02	1.67E-01
F25	1.39E-20	1.13E-20	4.03E-19	1.95E-03	1.24E-07	4.03E-19	4.03E-19	1.39E-20	4.03E-19
F26	1.63E-01	6.01E-02	1.35E-04	5.38E-15	1.06E-05	5.26E-13	2.15E-01	1.43E-13	5.85E-06
F27	3.23E-17	1.88E-17	2.09E-16	1.07E-13	1.07E-13	2.92E-14	7.77E-15	5.03E-16	1.07E-13
F28	7.08E-01	3.62E-02	1.72E-02	4.73E-12	8.83E-06	2.09E-11	4.74E-09	7.09E-18	4.73E-08
F29	1.63E-14	1.55E-14	8.26E-15	2.56E-15	9.41E-14	7.43E-15	2.21E-13	1.34E-15	1.55E-14

**Table A6:** Wilcoxon rank sum results of MSAO-EDA and its competitors on CEC2017 (30 D).

No.	SAO	EO	RIME	AFDBARO	CSOAOA	MRFO	EOSMA	JADE	CFOA
F1	3.72E-18	5.64E-17	3.30E-18	1.74E-11	3.30E-18	5.33E-17	5.33E-17	3.30E-18	3.30E-18
F2	2.31E-19	2.31E-19	2.31E-19	2.31E-19	2.31E-19	2.31E-19	2.31E-19	2.31E-19	2.31E-19
F3	1.79E-17	1.85E-15	4.47E-16	1.65E-12	3.30E-18	1.05E-11	1.21E-13	3.50E-18	3.30E-18
F4	9.46E-04	1.72E-07	4.53E-11	1.47E-16	1.74E-16	6.01E-15	1.95E-02	3.50E-18	1.08E-15
F5	3.30E-18	3.30E-18	3.30E-18	3.30E-18	3.30E-18	3.30E-18	3.30E-18	3.30E-18	3.30E-18
F6	8.80E-04	3.06E-01	3.06E-01	1.22E-09	7.96E-18	2.38E-07	7.18E-02	5.03E-17	1.09E-08
F7	3.84E-03	1.68E-08	1.18E-12	3.04E-16	1.47E-14	1.08E-15	1.36E-04	3.50E-18	8.71E-15
F8	1.39E-20	1.39E-20	1.39E-20	1.39E-20	1.39E-20	1.39E-20	1.39E-20	1.39E-20	1.39E-20
F9	2.19E-11	3.88E-06	1.07E-07	3.63E-06	2.38E-03	1.96E-05	2.56E-05	1.64E-10	5.37E-03
F10	1.78E-09	1.64E-10	1.13E-17	1.42E-17	3.30E-18	5.58E-16	3.01E-11	3.50E-18	3.30E-18
F11	3.30E-18	3.30E-18	3.30E-18	3.30E-18	3.30E-18	3.30E-18	3.30E-18	3.30E-18	3.30E-18
F12	3.30E-18	3.30E-18	3.30E-18	3.30E-18	3.30E-18	6.68E-18	3.50E-18	3.30E-18	3.30E-18
F13	3.30E-18	3.30E-18	3.30E-18	3.30E-18	3.30E-18	3.30E-18	3.30E-18	3.30E-18	3.30E-18
F14	3.30E-18	2.01E-17	3.30E-18	3.30E-18	3.30E-18	3.30E-18	3.30E-18	3.30E-18	3.30E-18
F15	3.45E-01	2.45E-02	6.58E-04	2.54E-10	9.60E-08	7.97E-09	6.02E-01	5.33E-17	5.05E-10
F16	1.49E-05	2.77E-03	9.67E-15	3.39E-16	2.30E-16	4.47E-16	1.29E-07	3.30E-18	4.49E-17
F17	3.30E-18	3.30E-18	3.30E-18	3.30E-18	3.30E-18	3.30E-18	3.30E-18	3.30E-18	3.30E-18
F18	3.30E-18	1.07E-17	3.30E-18	3.30E-18	3.30E-18	3.30E-18	4.99E-18	3.30E-18	3.30E-18
F19	3.39E-12	6.20E-11	2.18E-15	1.20E-17	7.07E-17	5.64E-17	5.96E-13	3.30E-18	4.70E-18
F20	1.77E-03	2.99E-06	3.64E-13	5.63E-17	4.26E-10	3.56E-14	1.28E-02	3.71E-18	1.91E-14
F21	3.13E-18	3.13E-18	3.13E-18	3.13E-18	3.13E-18	3.13E-18	3.13E-18	3.13E-18	3.13E-18
F22	2.18E-15	1.39E-16	3.94E-18	3.50E-18	3.30E-18	4.70E-18	1.30E-12	3.30E-18	3.30E-18
F23	1.57E-15	1.51E-17	3.50E-18	3.30E-18	3.30E-18	3.30E-18	2.21E-12	3.30E-18	3.30E-18
F24	5.79E-09	1.69E-02	6.31E-17	6.31E-17	3.30E-18	8.40E-07	2.67E-08	3.50E-18	3.30E-18
F25	1.39E-07	1.81E-13	2.33E-13	1.51E-09	5.14E-13	2.99E-06	6.58E-03	3.30E-18	9.43E-02
F26	1.09E-03	2.71E-06	3.00E-08	4.99E-18	6.93E-14	4.48E-16	1.38E-03	6.31E-17	6.68E-17
F27	1.81E-14	9.68E-11	3.78E-17	1.27E-13	3.30E-18	1.45E-11	4.55E-09	3.16E-09	4.70E-18
F28	5.73E-02	3.99E-01	1.66E-12	3.94E-18	1.07E-17	2.61E-14	1.45E-05	3.30E-18	3.30E-18
F29	3.30E-18	4.73E-16	3.30E-18	3.30E-18	3.30E-18	3.30E-18	3.30E-18	3.30E-18	3.30E-18

**Table A7:** Wilcoxon rank sum results of MSAO-EDA and its competitors on CEC2017 (50 D).

No.	SAO	EO	RIME	AFDBARO	CSOAOA	MRFO	EOSMA	JADE	CFOA
F1	3.72E-18	5.64E-17	3.30E-18	1.74E-11	3.30E-18	5.33E-17	5.33E-17	3.30E-18	3.30E-18
F2	2.31E-19	2.31E-19	2.31E-19	2.31E-19	2.31E-19	2.31E-19	2.31E-19	2.31E-19	2.31E-19
F3	1.79E-17	1.85E-15	4.47E-16	1.65E-12	3.30E-18	1.05E-11	1.21E-13	3.50E-18	3.30E-18
F4	9.46E-04	1.72E-07	4.53E-11	1.47E-16	1.74E-16	6.01E-15	1.95E-02	3.50E-18	1.08E-15
F5	3.30E-18	3.30E-18	3.30E-18	3.30E-18	3.30E-18	3.30E-18	3.30E-18	3.30E-18	3.30E-18
F6	8.80E-04	3.06E-01	3.06E-01	1.22E-09	7.96E-18	2.38E-07	7.18E-02	5.03E-17	1.09E-08
F7	3.84E-03	1.68E-08	1.18E-12	3.04E-16	1.47E-14	1.08E-15	1.36E-04	3.50E-18	8.71E-15
F8	1.39E-20	1.39E-20	1.39E-20	1.39E-20	1.39E-20	1.39E-20	1.39E-20	1.39E-20	1.39E-20
F9	2.19E-11	3.88E-06	1.07E-07	3.63E-06	2.38E-03	1.96E-05	2.56E-05	1.64E-10	5.37E-03
F10	1.78E-09	1.64E-10	1.13E-17	1.42E-17	3.30E-18	5.58E-16	3.01E-11	3.50E-18	3.30E-18
F11	3.30E-18	3.30E-18	3.30E-18	3.30E-18	3.30E-18	3.30E-18	3.30E-18	3.30E-18	3.30E-18
F12	3.30E-18	3.30E-18	3.30E-18	3.30E-18	3.30E-18	6.68E-18	3.50E-18	3.30E-18	3.30E-18
F13	3.30E-18	3.30E-18	3.30E-18	3.30E-18	3.30E-18	3.30E-18	3.30E-18	3.30E-18	3.30E-18
F14	3.30E-18	2.01E-17	3.30E-18	3.30E-18	3.30E-18	3.30E-18	3.30E-18	3.30E-18	3.30E-18
F15	3.45E-01	2.45E-02	6.58E-04	2.54E-10	9.60E-08	7.97E-09	6.02E-01	5.33E-17	5.05E-10
F16	1.49E-05	2.77E-03	9.67E-15	3.39E-16	2.30E-16	4.47E-16	1.29E-07	3.30E-18	4.49E-17
F17	3.30E-18	3.30E-18	3.30E-18	3.30E-18	3.30E-18	3.30E-18	3.30E-18	3.30E-18	3.30E-18
F18	3.30E-18	1.07E-17	3.30E-18	3.30E-18	3.30E-18	3.30E-18	4.99E-18	3.30E-18	3.30E-18
F19	3.39E-12	6.20E-11	2.18E-15	1.20E-17	7.07E-17	5.64E-17	5.96E-13	3.30E-18	4.70E-18
F20	1.77E-03	2.99E-06	3.64E-13	5.63E-17	4.26E-10	3.56E-14	1.28E-02	3.71E-18	1.91E-14
F21	3.13E-18	3.13E-18	3.13E-18	3.13E-18	3.13E-18	3.13E-18	3.13E-18	3.13E-18	3.13E-18
F22	2.18E-15	1.39E-16	3.94E-18	3.50E-18	3.30E-18	4.70E-18	1.30E-12	3.30E-18	3.30E-18
F23	1.57E-15	1.51E-17	3.50E-18	3.30E-18	3.30E-18	3.30E-18	2.21E-12	3.30E-18	3.30E-18
F24	5.79E-09	1.69E-02	6.31E-17	6.31E-17	3.30E-18	8.40E-07	2.67E-08	3.50E-18	3.30E-18
F25	1.39E-07	1.81E-13	2.33E-13	1.51E-09	5.14E-13	2.99E-06	6.58E-03	3.30E-18	9.43E-02
F26	1.09E-03	2.71E-06	3.00E-08	4.99E-18	6.93E-14	4.48E-16	1.38E-03	6.31E-17	6.68E-17
F27	1.81E-14	9.68E-11	3.78E-17	1.27E-13	3.30E-18	1.45E-11	4.55E-09	3.16E-09	4.70E-18
F28	5.73E-02	3.99E-01	1.66E-12	3.94E-18	1.07E-17	2.61E-14	1.45E-05	3.30E-18	3.30E-18
F29	3.30E-18	4.73E-16	3.30E-18	3.30E-18	3.30E-18	3.30E-18	3.30E-18	3.30E-18	3.30E-18

**Table A8:** Wilcoxon rank sum results of MSAO-EDA and its competitors on CEC2017 (100 D).

No.	SAO	EO	RIME	AFDBARO	CSOAOA	MRFO	EOSMA	JADE	CFOA
F1	3.72E-18	5.64E-17	3.30E-18	1.74E-11	3.30E-18	5.33E-17	5.33E-17	3.30E-18	3.30E-18
F2	2.31E-19	2.31E-19	2.31E-19	2.31E-19	2.31E-19	2.31E-19	2.31E-19	2.31E-19	2.31E-19
F3	1.79E-17	1.85E-15	4.47E-16	1.65E-12	3.30E-18	1.05E-11	1.21E-13	3.50E-18	3.30E-18
F4	9.46E-04	1.72E-07	4.53E-11	1.47E-16	1.74E-16	6.01E-15	1.95E-02	3.50E-18	1.08E-15
F5	3.30E-18	3.30E-18	3.30E-18	3.30E-18	3.30E-18	3.30E-18	3.30E-18	3.30E-18	3.30E-18
F6	8.80E-04	3.06E-01	3.06E-01	1.22E-09	7.96E-18	2.38E-07	7.18E-02	5.03E-17	1.09E-08
F7	3.84E-03	1.68E-08	1.18E-12	3.04E-16	1.47E-14	1.08E-15	1.36E-04	3.50E-18	8.71E-15
F8	1.39E-20	1.39E-20	1.39E-20	1.39E-20	1.39E-20	1.39E-20	1.39E-20	1.39E-20	1.39E-20
F9	2.19E-11	3.88E-06	1.07E-07	3.63E-06	2.38E-03	1.96E-05	2.56E-05	1.64E-10	5.37E-03
F10	1.78E-09	1.64E-10	1.13E-17	1.42E-17	3.30E-18	5.58E-16	3.01E-11	3.50E-18	3.30E-18
F11	3.30E-18	3.30E-18	3.30E-18	3.30E-18	3.30E-18	3.30E-18	3.30E-18	3.30E-18	3.30E-18
F12	3.30E-18	3.30E-18	3.30E-18	3.30E-18	3.30E-18	6.68E-18	3.50E-18	3.30E-18	3.30E-18
F13	3.30E-18	3.30E-18	3.30E-18	3.30E-18	3.30E-18	3.30E-18	3.30E-18	3.30E-18	3.30E-18
F14	3.30E-18	2.01E-17	3.30E-18	3.30E-18	3.30E-18	3.30E-18	3.30E-18	3.30E-18	3.30E-18
F15	3.45E-01	2.45E-02	6.58E-04	2.54E-10	9.60E-08	7.97E-09	6.02E-01	5.33E-17	5.05E-10
F16	1.49E-05	2.77E-03	9.67E-15	3.39E-16	2.30E-16	4.47E-16	1.29E-07	3.30E-18	4.49E-17
F17	3.30E-18	3.30E-18	3.30E-18	3.30E-18	3.30E-18	3.30E-18	3.30E-18	3.30E-18	3.30E-18
F18	3.30E-18	1.07E-17	3.30E-18	3.30E-18	3.30E-18	3.30E-18	4.99E-18	3.30E-18	3.30E-18
F19	3.39E-12	6.20E-11	2.18E-15	1.20E-17	7.07E-17	5.64E-17	5.96E-13	3.30E-18	4.70E-18
F20	1.77E-03	2.99E-06	3.64E-13	5.63E-17	4.26E-10	3.56E-14	1.28E-02	3.71E-18	1.91E-14
F21	3.13E-18	3.13E-18	3.13E-18	3.13E-18	3.13E-18	3.13E-18	3.13E-18	3.13E-18	3.13E-18
F22	2.18E-15	1.39E-16	3.94E-18	3.50E-18	3.30E-18	4.70E-18	1.30E-12	3.30E-18	3.30E-18
F23	1.57E-15	1.51E-17	3.50E-18	3.30E-18	3.30E-18	3.30E-18	2.21E-12	3.30E-18	3.30E-18
F24	5.79E-09	1.69E-02	6.31E-17	6.31E-17	3.30E-18	8.40E-07	2.67E-08	3.50E-18	3.30E-18
F25	1.39E-07	1.81E-13	2.33E-13	1.51E-09	5.14E-13	2.99E-06	6.58E-03	3.30E-18	9.43E-02
F26	1.09E-03	2.71E-06	3.00E-08	4.99E-18	6.93E-14	4.48E-16	1.38E-03	6.31E-17	6.68E-17
F27	1.81E-14	9.68E-11	3.78E-17	1.27E-13	3.30E-18	1.45E-11	4.55E-09	3.16E-09	4.70E-18
F28	5.73E-02	3.99E-01	1.66E-12	3.94E-18	1.07E-17	2.61E-14	1.45E-05	3.30E-18	3.30E-18
F29	3.30E-18	4.73E-16	3.30E-18	3.30E-18	3.30E-18	3.30E-18	3.30E-18	3.30E-18	3.30E-18

**Table A9:** Comparison results on of MSAO-EDA and its competitors on CEC2022 (10 D).

No.	Metric	MSAO-EDA	SAO	EO	RIME	AFDBARO	CSOAOA	MRFO	EOSMA	JADE	CFOA
F1	Best	3.000E+02	3.000E+02	3.000E+02	3.000E+02	3.443E+02	3.051E+02	3.000E+02	3.000E+02	6.279E+02	3.018E+02
	Mean	3.000E+02	3.000E+02	3.000E+02	3.001E+02	5.891E+02	5.312E+02	3.004E+02	3.000E+02	1.110E+03	5.889E+02
	Std	0.000E+00	9.651E-11	1.231E-06	7.523E-02	1.556E+02	2.234E+02	4.597E-01	6.460E-04	2.600E+02	2.173E+02
	Rank	1	2	3	5	9	7	6	4	10	8
F2	Best	4.000E+02	4.000E+02	4.003E+02	4.000E+02	4.000E+02	4.000E+02	4.000E+02	4.000E+02	4.000E+02	4.000E+02
	Mean	4.030E+02	4.069E+02	4.077E+02	4.073E+02	4.040E+02	4.010E+02	4.068E+02	4.015E+02	4.003E+02	4.002E+02
	Std	3.708E+00	2.984E+00	2.125E+00	1.019E+01	2.829E+00	2.245E+00	1.643E+01	2.614E+00	8.836E-01	1.739E-01
	Rank	5	8	10	9	6	3	7	4	2	1
F3	Best	6.000E+02	6.000E+02	6.000E+02	6.000E+02	6.000E+02	6.002E+02	6.000E+02	6.000E+02	6.000E+02	6.002E+02
	Mean	6.000E+02	6.000E+02	6.000E+02	6.001E+02	6.000E+02	6.006E+02	6.000E+02	6.000E+02	6.000E+02	6.017E+02
	Std	0.000E+00	1.364E-07	1.044E-06	4.679E-02	1.444E-02	3.337E-01	4.155E-04	1.935E-04	4.630E-03	8.697E-01
	Rank	1	2	3	8	6	9	4	5	7	10
F4	Best	8.020E+02	8.050E+02	8.020E+02	8.040E+02	8.040E+02	8.034E+02	8.060E+02	8.000E+02	8.180E+02	8.010E+02
	Mean	8.059E+02	8.133E+02	8.098E+02	8.161E+02	8.171E+02	8.084E+02	8.182E+02	8.068E+02	8.258E+02	8.084E+02
	Std	2.705E+00	4.312E+00	4.497E+00	7.541E+00	6.579E+00	2.405E+00	7.293E+00	3.274E+00	3.695E+00	3.631E+00
	Rank	1	6	5	7	8	3	9	2	10	4
F5	Best	9.000E+02	9.000E+02	9.000E+02	9.000E+02	9.002E+02	9.001E+02	9.000E+02	9.000E+02	9.000E+02	9.000E+02
	Mean	9.000E+02	9.000E+02	9.000E+02	9.001E+02	9.006E+02	9.011E+02	9.001E+02	9.000E+02	9.000E+02	9.005E+02
	Std	0.000E+00	1.080E-01	1.755E-02	1.166E-01	3.899E-01	1.349E+00	2.142E-01	1.371E-11	4.995E-04	3.458E-01
	Rank	1	5	4	6	9	10	7	2	3	8
F6	Best	1.800E+03	1.809E+03	1.854E+03	1.873E+03	1.807E+03	1.801E+03	1.853E+03	1.848E+03	1.919E+03	1.834E+03
	Mean	1.800E+03	5.468E+03	4.299E+03	4.002E+03	1.848E+03	1.813E+03	2.972E+03	1.969E+03	2.388E+03	2.621E+03
	Std	2.450E-01	2.417E+03	2.111E+03	1.909E+03	1.743E+01	6.930E+00	1.194E+03	6.996E+01	3.325E+02	7.682E+02
	Rank	1	10	9	8	3	2	7	4	5	6
F7	Best	2.000E+03	2.001E+03	2.000E+03	2.000E+03	2.000E+03	2.010E+03	2.000E+03	2.000E+03	2.009E+03	2.011E+03
	Mean	2.011E+03	2.017E+03	2.019E+03	2.012E+03	2.010E+03	2.017E+03	2.010E+03	2.006E+03	2.015E+03	2.030E+03
	Std	1.015E+01	7.986E+00	5.943E+00	9.648E+00	8.772E+00	4.170E+00	9.369E+00	7.767E+00	2.873E+00	7.353E+00
	Rank	4	7	9	5	2	8	3	1	6	10
F8	Best	2.201E+03	2.200E+03	2.201E+03	2.201E+03	2.201E+03	2.206E+03	2.201E+03	2.201E+03	2.210E+03	2.203E+03
	Mean	2.212E+03	2.215E+03	2.215E+03	2.214E+03	2.218E+03	2.213E+03	2.220E+03	2.206E+03	2.218E+03	2.214E+03
	Std	9.278E+00	8.683E+00	8.968E+00	9.533E+00	5.287E+00	3.932E+00	7.736E+00	4.436E+00	3.449E+00	7.924E+00
	Rank	2	7	6	4	9	3	10	1	8	5
F9	Best	2.529E+03	2.529E+03	2.529E+03	2.529E+03	2.529E+03	2.529E+03	2.529E+03	2.529E+03	2.529E+03	2.529E+03
	Mean	2.529E+03	2.529E+03	2.529E+03	2.529E+03	2.529E+03	2.530E+03	2.529E+03	2.529E+03	2.529E+03	2.529E+03
	Std	0.000E+00	0.000E+00	0.000E+00	1.269E-03	1.229E-02	7.449E-01	4.595E-03	1.104E-11	3.184E-04	2.359E-01
	Rank	1	1	1	5	7	10	8	4	6	9
F10	Best	2.500E+03	2.500E+03	2.500E+03	2.500E+03	2.500E+03	2.500E+03	2.500E+03	2.500E+03	2.500E+03	2.500E+03
	Mean	2.500E+03	2.500E+03	2.502E+03	2.500E+03	2.500E+03	2.500E+03	2.500E+03	2.500E+03	2.500E+03	2.500E+03
	Std	3.428E-02	4.639E-02	1.619E+01	7.766E-02	9.662E-02	7.005E-02	8.424E-02	4.246E-02	4.242E-02	5.934E-02
	Rank	2	4	10	5	9	7	6	1	8	3
F11	Best	2.600E+03	2.600E+03	2.600E+03	2.602E+03	2.600E+03	2.618E+03	2.600E+03	2.600E+03	2.600E+03	2.605E+03
	Mean	2.612E+03	2.641E+03	2.753E+03	2.805E+03	2.630E+03	2.756E+03	2.673E+03	2.630E+03	2.736E+03	2.846E+03
	Std	5.881E+01	1.043E+02	1.515E+02	1.616E+02	4.561E+01	1.349E+02	1.565E+02	9.006E+01	1.469E+02	1.239E+02
	Rank	1	4	7	9	2	8	5	3	6	10
F12	Best	2.859E+03	2.859E+03	2.860E+03	2.859E+03	2.862E+03	2.859E+03	2.859E+03	2.859E+03	2.863E+03	2.859E+03
	Mean	2.862E+03	2.862E+03	2.862E+03	2.863E+03	2.866E+03	2.863E+03	2.867E+03	2.860E+03	2.865E+03	2.863E+03
	Std	2.019E+00	1.414E+00	1.498E+00	2.053E+00	1.678E+00	1.611E+00	2.005E+00	1.485E+00	4.067E-01	1.194E+00
	Rank	4	3	2	6	9	5	10	1	8	7

**Table A10:** Comparison results on of MSAO-EDA and its competitors on CEC2022 (20 D).

No.	Metric	MSAO-EDA	SAO	EO	RIME	AFDBARO	CSOAOA	MRFO	EOSMA	JADE	CFOA
F1	Best	3.000E+02	3.004E+02	3.001E+02	3.013E+02	2.799E+03	1.820E+03	6.450E+02	3.019E+02	7.030E+03	1.695E+03
	Mean	3.000E+02	3.201E+02	3.009E+02	3.065E+02	7.444E+03	5.411E+03	1.373E+03	3.167E+02	1.098E+04	4.001E+03
	Std	0.000E+00	2.663E+01	5.519E-01	3.796E+00	2.767E+03	1.923E+03	4.188E+02	1.125E+01	1.564E+03	1.793E+03
	Rank	1	5	2	3	9	8	6	4	10	7
F2	Best	4.005E+02	4.449E+02	4.449E+02	4.275E+02	4.009E+02	4.659E+02	4.025E+02	4.031E+02	4.491E+02	4.306E+02
	Mean	4.472E+02	4.524E+02	4.511E+02	4.522E+02	4.448E+02	4.998E+02	4.542E+02	4.481E+02	4.491E+02	4.655E+02
	Std	6.890E+00	8.531E+00	6.811E+00	1.148E+01	2.090E+01	2.885E+01	1.854E+01	6.713E+00	1.104E-04	1.128E+01
	Rank	2	7	5	6	1	10	8	3	4	9
F3	Best	6.000E+02	6.000E+02	6.000E+02	6.001E+02	6.000E+02	6.016E+02	6.000E+02	6.000E+02	6.001E+02	6.035E+02
	Mean	6.000E+02	6.000E+02	6.000E+02	6.004E+02	6.000E+02	6.037E+02	6.001E+02	6.000E+02	6.001E+02	6.082E+02
	Std	4.545E-08	4.213E-04	1.031E-04	1.382E-01	7.616E-02	1.157E+00	3.235E-01	2.514E-03	8.382E-03	2.298E+00
	Rank	1	3	2	8	5	9	7	4	6	10
F4	Best	8.070E+02	8.125E+02	8.129E+02	8.169E+02	8.382E+02	8.277E+02	8.378E+02	8.060E+02	8.718E+02	8.278E+02
	Mean	8.206E+02	8.285E+02	8.264E+02	8.407E+02	8.659E+02	8.444E+02	8.610E+02	8.184E+02	8.945E+02	8.432E+02
	Std	1.348E+01	1.373E+01	7.349E+00	1.290E+01	1.620E+01	6.760E+00	1.445E+01	6.554E+00	7.194E+00	8.558E+00
	Rank	2	4	3	5	9	7	8	1	10	6
F5	Best	9.000E+02	9.000E+02	9.000E+02	9.002E+02	9.059E+02	9.087E+02	9.001E+02	9.000E+02	9.000E+02	9.174E+02
	Mean	9.000E+02	9.001E+02	9.001E+02	9.050E+02	9.318E+02	1.025E+03	1.124E+03	9.003E+02	9.000E+02	9.533E+02
	Std	0.000E+00	1.261E-01	1.947E-01	9.171E+00	3.571E+01	1.299E+02	3.388E+02	3.018E-01	1.037E-02	2.404E+01
	Rank	1	3	4	6	7	9	10	5	2	8
F6	Best	1.800E+03	1.834E+03	1.869E+03	2.561E+03	1.924E+03	2.069E+03	1.863E+03	1.958E+03	4.717E+05	2.104E+03
	Mean	1.804E+03	5.861E+03	5.015E+03	9.264E+03	2.381E+03	6.757E+03	5.630E+03	6.025E+03	1.260E+06	2.833E+03
	Std	2.299E+00	5.904E+03	4.634E+03	5.391E+03	5.254E+02	8.570E+03	4.791E+03	4.435E+03	4.302E+05	5.412E+02
	Rank	1	6	4	9	2	8	5	7	10	3
F7	Best	2.020E+03	2.020E+03	2.007E+03	2.022E+03	2.024E+03	2.029E+03	2.011E+03	2.022E+03	2.045E+03	2.032E+03
	Mean	2.030E+03	2.038E+03	2.030E+03	2.035E+03	2.045E+03	2.044E+03	2.046E+03	2.029E+03	2.069E+03	2.063E+03
	Std	9.734E+00	1.217E+01	1.092E+01	1.019E+01	1.468E+01	6.847E+00	1.941E+01	5.010E+00	7.194E+00	1.389E+01
	Rank	2	5	3	4	7	6	8	1	10	9
F8	Best	2.229E+03	2.220E+03	2.221E+03	2.222E+03	2.221E+03	2.224E+03	2.221E+03	2.220E+03	2.230E+03	2.223E+03
	Mean	2.233E+03	2.223E+03	2.224E+03	2.225E+03	2.223E+03	2.226E+03	2.227E+03	2.226E+03	2.235E+03	2.227E+03
	Std	1.705E+00	2.213E+00	2.043E+00	1.490E+00	3.164E+00	7.815E-01	1.755E+01	1.392E+00	1.670E+00	1.509E+00
	Rank	9	1	3	4	2	6	7	5	10	8
F9	Best	2.481E+03	2.481E+03	2.481E+03	2.481E+03	2.481E+03	2.483E+03	2.481E+03	2.481E+03	2.481E+03	2.481E+03
	Mean	2.481E+03	2.481E+03	2.481E+03	2.481E+03	2.481E+03	2.489E+03	2.481E+03	2.481E+03	2.481E+03	2.485E+03
	Std	4.547E-13	1.549E-05	1.693E-04	8.528E-02	8.321E-03	4.372E+00	1.021E-01	2.835E-06	7.954E-03	2.402E+00
	Rank	1	3	4	7	5	10	8	2	6	9
F10	Best	2.500E+03	2.500E+03	2.500E+03	2.426E+03	2.419E+03	2.500E+03	2.500E+03	2.500E+03	2.500E+03	2.500E+03
	Mean	2.505E+03	2.503E+03	2.556E+03	2.518E+03	2.488E+03	2.501E+03	2.519E+03	2.500E+03	2.500E+03	2.500E+03
	Std	2.486E+01	1.860E+01	1.721E+02	4.953E+01	2.548E+01	1.693E-01	5.213E+01	5.275E-02	5.485E-02	8.785E-02
	Rank	7	6	10	8	1	5	9	2	4	3
F11	Best	2.900E+03	2.900E+03	2.900E+03	2.925E+03	2.747E+03	3.117E+03	2.600E+03	2.900E+03	2.900E+03	3.041E+03
	Mean	2.900E+03	2.900E+03	2.900E+03	2.956E+03	2.959E+03	3.271E+03	2.932E+03	2.902E+03	2.900E+03	3.219E+03
	Std	2.572E-13	3.916E-03	4.171E-10	1.580E+01	6.690E+01	7.889E+01	6.878E+01	1.400E+01	2.887E-02	8.996E+01
	Rank	1	3	2	7	8	10	6	5	4	9
F12	Best	2.931E+03	2.934E+03	2.933E+03	2.934E+03	2.959E+03	2.941E+03	2.947E+03	2.931E+03	2.943E+03	2.939E+03
	Mean	2.942E+03	2.943E+03	2.943E+03	2.944E+03	2.991E+03	2.950E+03	2.975E+03	2.939E+03	2.952E+03	2.957E+03
	Std	9.295E+00	6.330E+00	6.367E+00	5.262E+00	2.178E+01	5.825E+00	3.018E+01	4.434E+00	3.804E+00	6.372E+00
	Rank	2	4	3	5	10	6	9	1	7	8

**Table A11:** Wilcoxon rank sum results of MSAO-EDA and its competitors on CEC2022 (10 D).

No.	SAO	EO	RIME	AFDBARO	CSOAOA	MRFO	EOSMA	JADE	CFOA
F1	3.72E-18	5.64E-17	3.30E-18	1.74E-11	3.30E-18	5.33E-17	5.33E-17	3.30E-18	3.30E-18
F2	2.31E-19	2.31E-19	2.31E-19	2.31E-19	2.31E-19	2.31E-19	2.31E-19	2.31E-19	2.31E-19
F3	1.79E-17	1.85E-15	4.47E-16	1.65E-12	3.30E-18	1.05E-11	1.21E-13	3.50E-18	3.30E-18
F4	9.46E-04	1.72E-07	4.53E-11	1.47E-16	1.74E-16	6.01E-15	1.95E-02	3.50E-18	1.08E-15
F5	3.30E-18	3.30E-18	3.30E-18	3.30E-18	3.30E-18	3.30E-18	3.30E-18	3.30E-18	3.30E-18
F6	8.80E-04	3.06E-01	3.06E-01	1.22E-09	7.96E-18	2.38E-07	7.18E-02	5.03E-17	1.09E-08
F7	3.84E-03	1.68E-08	1.18E-12	3.04E-16	1.47E-14	1.08E-15	1.36E-04	3.50E-18	8.71E-15
F8	1.39E-20	1.39E-20	1.39E-20	1.39E-20	1.39E-20	1.39E-20	1.39E-20	1.39E-20	1.39E-20
F9	2.19E-11	3.88E-06	1.07E-07	3.63E-06	2.38E-03	1.96E-05	2.56E-05	1.64E-10	5.37E-03
F10	1.78E-09	1.64E-10	1.13E-17	1.42E-17	3.30E-18	5.58E-16	3.01E-11	3.50E-18	3.30E-18
F11	3.30E-18	3.30E-18	3.30E-18	3.30E-18	3.30E-18	3.30E-18	3.30E-18	3.30E-18	3.30E-18
F12	3.30E-18	3.30E-18	3.30E-18	3.30E-18	3.30E-18	6.68E-18	3.50E-18	3.30E-18	3.30E-18

**Table A12:** Wilcoxon rank sum results of MSAO-EDA and its competitors on CEC2022 (20 D).

No.	SAO	EO	RIME	AFDBARO	CSOAOA	MRFO	EOSMA	JADE	CFOA
F1	1.39E-20	1.39E-20	1.39E-20	1.39E-20	1.39E-20	1.39E-20	1.39E-20	1.39E-20	1.39E-20
F2	3.53E-16	3.68E-17	4.63E-11	7.62E-01	3.24E-19	3.79E-09	4.69E-14	3.24E-19	1.22E-15
F3	3.30E-18	3.30E-18	3.30E-18	3.30E-18	3.30E-18	3.30E-18	3.30E-18	3.30E-18	3.30E-18
F4	4.27E-06	4.70E-06	1.07E-12	2.18E-16	6.34E-15	3.79E-16	5.69E-01	4.43E-18	1.81E-14
F5	1.39E-20	1.39E-20	1.39E-20	1.39E-20	1.39E-20	1.39E-20	1.39E-20	1.39E-20	1.39E-20
F6	3.30E-18	3.30E-18	3.30E-18	3.30E-18	3.30E-18	3.30E-18	3.30E-18	3.30E-18	3.30E-18
F7	5.29E-05	6.68E-01	1.07E-03	5.13E-09	4.30E-12	1.89E-08	3.99E-01	2.26E-17	5.89E-16
F8	3.30E-18	5.60E-18	3.30E-18	1.14E-15	3.30E-18	2.42E-15	3.30E-18	5.99E-10	7.51E-18
F9	1.99E-20	1.98E-20	1.99E-20	1.99E-20	1.99E-20	1.99E-20	1.99E-20	1.99E-20	1.99E-20
F10	2.33E-10	1.14E-06	7.03E-01	1.03E-05	2.18E-15	5.17E-06	1.10E-13	6.06E-06	4.84E-03
F11	3.93E-19	3.93E-19	3.93E-19	8.21E-12	3.93E-19	8.84E-18	3.93E-19	3.93E-19	3.93E-19
F12	2.87E-01	4.22E-01	1.72E-02	1.90E-17	4.93E-09	3.04E-16	4.61E-02	4.22E-13	4.15E-14
